# Supplementary material for: Developing a priority global research agenda for antimicrobial resistance in the human health sector: protocol for a scoping review
Source: BMJ Open. 2022 Jun 2;12(6):e060553. doi: 10.1136/bmjopen-2021-060553 (PMC9163534; doi:10.1136/bmjopen-2021-060553)
Supplement: Supplementary data [file bmjopen-2021-060553supp001.pdf]

Supplemental material

Supplementary Table 1. Full search terms for the bibliographic databases

Embase
http://www.embase.com

#		Results 10/12/2021
**1** – AMR	('drug resistance'/de OR 'antibiotic resistance'/exp OR 'antifungal resistance'/exp OR 'cross resistance'/exp OR 'extensive drug resistance'/exp OR 'multidrug resistance'/exp OR (drug-resistan* OR multidrug-resistan* OR anti-biotic-resistan* OR antibiotic-resistan* OR antibacterial-resistan* OR anti-bacterial-resistan* OR bacterial-resistan* OR bacterium-resistan* OR antimicrobial-resistan* OR microbial-resistan* OR anti-fungal-resistan* OR antifungal-resistan* OR cross-resistan* OR antibiotic-non-susceptib* OR antibiotic-nonsusceptib* OR drug-non-susceptib* OR drug-nonsusceptib*):ab,ti)<br>OR<br>('carbapenem resistant Acinetobacter baumannii'/exp OR ((Acinetobacter-baumannii* OR Achromobacter-mucosus OR Acinetobacter-baumanii OR Acinetobacter-baumanni OR Acinetobacter-genomosp.-2 OR Acinetobacter-genomospecies-2 OR Bacterium-anitratum OR Bact.-anitratum) AND (carbapenem-resistan* OR carbapenem-non-susceptib* OR carbapenem-nonsuscept*)):ti,ab)<br>OR<br>('carbapenem resistant Pseudomonas aeruginosa'/exp OR ((Pseudomonas-aeruginosa OR Bacillus-aeruginosus OR Bacillus-pyocyaneus OR Bacterium-aeruginosum OR Bacterium-pyocyaneum OR blue-pus-organism OR Micrococcus-pyocyaneus OR P.-aeruginosa OR Pseudomonas-polycolor OR Pseudomonas-pyoceaneus OR Pseudomonas-pyocyanea OR Pseudomonas-pyocyaneus) AND (carbapenem-resistan* OR carbapenem-non-susceptib* OR carbapenem-nonsuscept*)):ti,ab)<br>OR<br>('carbapenem-resistant Enterobacteriaceae'/exp OR ((Enterobacteriaceae OR enteric-bacteria OR enterobacteria OR enterobacteriacea OR enterobacterium OR Klebsiella-pneumonia OR Escherichia-coli OR Bacillus-coli OR Bacillus-escherichii OR Bacterium-coli OR Bacterium-coli OR bacterium-E3 OR coli-bacillus OR coli-bacterium OR colibacillus OR colon-bacillus OR E-coli OR E.-coli OR E.coli OR Enterococcus-coli OR Escherichia-alkalescens OR Enterobacter OR Serratia OR Proteus OR Providencia OR Morganella) AND (carbapenem-resistan* OR carbapenem-non-susceptib* OR carbapenem-nonsuscept* OR 3rd-generation-cephalosporin-resistan* OR third-generation-cephalosporin-resistan* OR third-generation-cephalosporin-non-susceptib*)):ti,ab)<br>OR	513,168

	('vancomycin resistant Enterococcus'/exp OR (VAN-R-enterococci OR VAN-R-Enterococcus OR (Streptococcus-faecium OR Enterococcus-faecium) AND (vancomycin-resistan* OR VAN-resistan* OR vancomycin-non-susceptib* OR vancomycin-nonsusceptib*)):ti,ab)<br>OR<br>('methicillin resistant Staphylococcus aureus'/exp OR 'methicillin resistant Staphylococcus aureus infection'/exp OR 'vancomycin resistant Staphylococcus aureus'/exp OR 'vancomycin intermediate Staphylococcus aureus'/exp OR (MRSA OR VRSA OR (Staphylococcus-aureus OR S.-aureus) AND (vancomycin-resistan* OR VAN-resistan* OR vancomycin-nonsusceptib* OR vancomycin-non-susceptib* OR Van-intermediate-resistan* OR vancomycin-intermediate-resistan* OR methicillin-resistan* OR methicillin-nonsusceptib* OR methicillin-non-susceptib*)):ti,ab)<br>OR<br>(((Helicobacter-pylori OR Campylobacter-pylori* OR Helicobacter-nemestrinae) AND (clarithromycin-resistan* OR clarithromycin-nonsusceptible OR clarithromycin-non-susceptib*)):ti,ab)<br>OR<br>(((Campylobacter) AND (fluoroquinolone-resistan* OR fluorinated-quinolone-resistan* OR fluoro-quinolone-resistan* OR fluoroquinolones-resistan* OR fluoroquinolone-nonsusceptible OR fluoroquinolone-non-susceptible)):ti,ab)<br>OR<br>(((Salmonella) AND (fluoroquinolone-resistan* OR fluorinated-quinolone-resistan* OR fluoro-quinolone-resistan* OR fluoroquinolones-resistan* OR fluoroquinolone-nonsusceptible OR fluoroquinolone-non-susceptible)):ti,ab)<br>OR<br>(((Neisseria-gonorrhoeae OR Gonococcus-neisseri OR Gonococcus-neisseria OR Micrococcus-gonorrhoeae OR N.-gonorrhoeae OR Neisseria-gonococcus OR Neisseria-gonorrhaeae OR Neisseria-gonorrhea OR Neisseria-gonorrheae OR Neisseria-gonorrhoea) AND (3rd-generation-cephalosporin-resistan* OR third-generation-cephalosporin-resistan* OR fluoroquinolone-resistan* OR fluorinated-quinolone-resistan* OR fluoro-quinolone-resistan* OR fluoroquinolones-resistan* OR fluoroquinolone-nonsusceptible OR fluoroquinolone-non-susceptible)):ti,ab)<br>OR<br>('penicillin resistant Streptococcus pneumoniae'/exp OR ((Streptococcus-pneumoniae OR Diplococcus-pneumoniae OR Micrococcus-pneumoniae OR Pneumococcus OR pneumococcus OR Pneumococcus-pneumoniae) AND (penicillin-resistan* OR penicillin-non-susceptib* OR penicillin-nonsusceptib* OR fluoroquinolone-nonsusceptible OR fluoroquinolone-non-susceptible)):ti,ab)<br>OR	

	(((Haemophilus-influenza* OR Hemophilus-influenza* OR H.-influenza* OR Bacterium-influenza* OR Coccobacillus-pfeifferi OR Hemophilus-influenzae OR influenza-bacillus OR Mycobacterium-influenzae OR pfeiffer-bacillus) AND (ampicillin-resistan* OR Amp-resistan* OR ampicillin-nonsusceptib* OR ampicillin-non-susceptib*)):ti,ab)<br>OR<br>(((Shigella) AND (fluoroquinolone-resistan* OR fluorinated-quinolone-resistan* OR fluoro-quinolone-resistan* OR fluoroquinolones-resistan* OR fluoroquinolone-nonsusceptible OR fluoroquinolone-non-susceptible)):ti,ab)<br>OR<br>('multidrug resistant tuberculosis'/exp OR (RR-TB OR MDR-TB OR Pre-XDR-TB OR XDR-TB OR drug-resistant-TB OR multidrug-resistant-TB OR multidrug-resistant-tuberculos* OR drug-resistant-tuberculos* OR ((Mycobacterium-tuberculos* OR pulmonary-tuberculos* OR pulmonary-TB) AND (isoniazid-resistan* OR rifampicin-resistan* OR floroquinolone-resistan* OR levofloxacin-resistan* OR moxifloxacin-resistan* OR bedaquiline-resistan* OR linezolid-resistan* OR multidrug-resistan* OR drug-resistan*))):ab,ti)<br>OR<br>(((Aspergillus OR a.-fumigatus OR a.-flavus OR Neosartorya-fumigate OR Candida OR c.-auris OR c.-albicans) AND (resistan* OR non-susceptib* OR nonsusceptib*)):ti,ab)	
**#2 -** SR	('systematic review'/de OR 'meta-analysis'/de OR ((systematic* NEAR/3 review*) OR metaanaly* OR meta-analy* OR scoping):ab,ti)	564,680
**#3**	#1 AND #2	7,288
**#4 –** Animal Studies filter	NOT ('animal'/exp OR 'nonhuman'/exp NOT ('animal'/exp OR 'nonhuman'/exp AND 'human'/exp))	6,949
**#5** Specific animals	NOT (rat OR rats OR mouse OR mice OR hamster OR hamsters OR dog OR dogs OR cat OR cats OR bovine OR sheep):ti	6946
**#6-** Date filter	#5 AND [1-1-2012]/sd NOT [2-1-2022]/sd AND [2012-2022]/py	5,288

Pubmed
https://pubmed.ncbi.nlm.nih.gov/

#		Results 10/12/2021
**1** – AMR	("Drug Resistance, Bacterial"[Mesh] OR "Drug Resistance, Fungal"[Mesh] OR "Drug Resistance, Multiple, Bacterial"[Mesh] OR "Drug Resistance, Multiple, Fungal"[Mesh] OR "Antimicrobial Stewardship"[Mesh] OR drug-resistan*[tiab] OR multidrug-	318,242

	resistan*[tiab] OR anti-biotic-resistan*[tiab] OR antibiotic-resistan*[tiab] OR antibacterial-resistan*[tiab] OR anti-bacterial-resistan*[tiab] OR bacterial-resistan*[tiab] OR bacterium-resistan*[tiab] OR antimicrobial-resistan*[tiab] OR microbial-resistan*[tiab] OR anti-fungal-resistan*[tiab] OR antifungal-resistan*[tiab] OR cross-resistan*[tiab] OR antibiotic-non-susceptib*[tiab] OR antibiotic-nonsusceptib*[tiab] OR drug-non-susceptib*[tiab] OR drug-nonsusceptib*[tiab])<br>OR<br>((("Acinetobacter baumannii"[Mesh] OR Acinetobacter-baumannii*[tiab] OR Achromobacter-mucosus[tiab] OR Acinetobacter-baumanii[tiab] OR Acinetobacter-baumanni[tiab] OR Acinetobacter-genomosp.-2[tiab] OR Acinetobacter-genomospecies-2[tiab] OR Bacterium-anitratum[tiab] OR Bact.-anitratum[tiab]) AND (carbapenem-resistan*[tiab] OR carbapenem-non-susceptib*[tiab] OR carbapenem-nonsuscept*[tiab])))<br>OR<br>((("Pseudomonas aeruginosa"[Mesh] OR Pseudomonas-aeruginosa[tiab] OR Bacillus-aeruginosus[tiab] OR Bacillus-pyocyaneus[tiab] OR Bacterium-aeruginosum[tiab] OR Bacterium-pyocyaneum[tiab] OR blue-pus-organism[tiab] OR Micrococcus-pyocyaneus[tiab] OR P.-aeruginosa[tiab] OR Pseudomonas-polycolor[tiab] OR Pseudomonas-pyoceaneus[tiab] OR Pseudomonas-pyocyanea[tiab] OR Pseudomonas-pyocyaneus[tiab]) AND (carbapenem-resistan*[tiab] OR carbapenem-non-susceptib*[tiab] OR carbapenem-nonsuscept*[tiab])))<br>OR<br>(("Carbapenem-Resistant Enterobacteriaceae"[Mesh] OR ("Enterobacteriaceae"[Mesh] OR Enterobacteriaceae[tiab] OR enteric-bacteria[tiab] OR enterobacteria[tiab] OR enterobacteriacea[tiab] OR enterobacterium[tiab] OR Klebsiella-pneumonia[tiab] OR Escherichia-coli[tiab] OR Bacillus-coli[tiab] OR Bacillus-escherichii[tiab] OR Bacterium-coli[tiab] OR Bacterium-coli[tiab] OR bacterium-E3[tiab] OR coli-bacillus[tiab] OR coli-bacterium[tiab] OR colibacillus[tiab] OR colon-bacillus[tiab] OR E-coli[tiab] OR E.-coli[tiab] OR E.coli[tiab] OR Enterococcus-coli[tiab] OR Escherichia-alkalescens[tiab] OR Enterobacter[tiab] OR Serratia[tiab] OR Proteus[tiab] OR Providencia[tiab] OR Morganella[tiab]) AND (carbapenem-resistan*[tiab] OR carbapenem-non-susceptib*[tiab] OR carbapenem-nonsuscept*[tiab] OR 3rd-generation-cephalosporin-resistan*[tiab] OR third-generation-cephalosporin-resistan*[tiab] OR third-generation-cephalosporin-non-susceptib*[tiab])))<br>OR<br>("Vancomycin-Resistant Enterococci"[Mesh] OR VAN-R-enterococci OR VAN-R-Enterococcus OR<br>(("Enterococcus faecium"[Mesh] OR Streptococcus-faecium OR Enterococcus-faecium) AND ("Vancomycin Resistance"[Mesh] OR	

	vancomycin-resistan*[tiab] OR VAN-resistan*[tiab] OR vancomycin-non-susceptib*[tiab] OR vancomycin-nonsusceptib*[tiab]))) OR ("Methicillin-Resistant Staphylococcus aureus"[Mesh] OR "vancomycin resistant Staphylococcus aureus"[mesh] OR "Vancomycin-Resistant Staphylococcus aureus"[Mesh] OR MRSA[tiab] OR VRSA[tiab] OR (("Staphylococcus aureus"[Mesh] OR Staphylococcus-aureus[tiab] OR S.-aureus[tiab]) AND ("Methicillin Resistance"[Mesh] OR "Vancomycin Resistance"[Mesh] OR vancomycin-resistan*[tiab] OR VAN-resistan*[tiab] OR vancomycin-nonsusceptib*[tiab] OR vancomycin-non-susceptib*[tiab] OR Van-intermediate-resistan*[tiab] OR vancomycin-intermediate-resistan*[tiab] OR methicillin-resistan*[tiab] OR methicillin-nonsusceptib*[tiab]))) OR ((("Helicobacter pylori"[Mesh] OR Helicobacter-pylori[tiab] OR Campylobacter-pylori*[tiab] OR Helicobacter-nemestrinae) AND (clarithromycin-resistan* OR clarithromycin-non-susceptib*[tiab]))) OR ((("Campylobacter"[Mesh] OR Campylobacter[tiab]) AND (fluoroquinolone-resistan*[tiab] OR fluorinated-quinolone-resistan*[tiab] OR fluoro-quinolone-resistan*[tiab] OR fluoroquinolones-resistan*[tiab] OR fluoroquinolone-nonsusceptible[tiab] OR fluoroquinolone-non-susceptible[tiab]))) OR ((("Salmonella"[Mesh] OR Salmonella[tiab]) AND (fluoroquinolone-resistan*[tiab] OR fluorinated-quinolone-resistan*[tiab] OR fluoro-quinolone-resistan*[tiab] OR fluoroquinolones-resistan*[tiab] OR fluoroquinolone-nonsusceptible[tiab] OR fluoroquinolone-non-susceptible[tiab]))) OR ((("Neisseria gonorrhoeae"[Mesh] OR Neisseria-gonorrhoeae[tiab] OR Gonococcus-neisseri[tiab] OR Gonococcus-neisseria[tiab] OR Micrococcus-gonorrhoeae[tiab] OR N.-gonorrhoeae[tiab] OR Neisseria-gonococcus[tiab] OR Neisseria-gonorrhaeae[tiab] OR Neisseria-gonorrhea[tiab] OR Neisseria-gonorrheae[tiab] OR Neisseria-gonorrhoea[tiab]) AND (3rd-generation-cephalosporin-resistan*[tiab] OR third-generation-cephalosporin-resistan*[tiab] OR fluoroquinolone-resistan*[tiab] OR fluorinated-quinolone-resistan*[tiab] OR fluoro-quinolone-resistan*[tiab] OR fluoroquinolones-resistan*[tiab] OR fluoroquinolone-nonsusceptible[tiab] OR fluoroquinolone-non-susceptible[tiab]))) OR ((("Streptococcus pneumoniae"[Mesh] OR Streptococcus-pneumoniae[tiab] OR Diplococcus-pneumoniae[tiab] OR Micrococcus-pneumoniae[tiab] OR Pneumococcus[tiab] OR pneumococcus[tiab] OR Pneumococcus-pneumoniae[tiab]) AND	

	("Penicillin Resistance"[Mesh] OR penicillin-resistan*[tiab] OR penicillin-non-susceptib*[tiab] OR penicillin-nonsusceptib*[tiab]))) OR ((("Haemophilus influenzae"[Mesh] OR Hemophilus-influenza*[tiab] OR Bacterium-influenzae OR Coccobacillus-pfeifferi OR H.-influenza*[tiab] OR Haemophilus-influenza*[tiab] OR influenza-bacillus[tiab] OR Mycobacterium-influenzae[tiab] OR pfeiffer-bacillus[tiab]) AND (ampicillin-resistan*[tiab] OR Amp-resistan*[tiab] OR ampicillin-nonsusceptib*[tiab] OR ampicillin-non-susceptib*[tiab]))) OR ((("Shigella"[Mesh] OR Shigella[tiab]) AND (fluoroquinolone-resistan*[tiab] OR fluorinated-quinolone-resistan*[tiab] OR fluoro-quinolone-resistan*[tiab] OR fluoroquinolones-resistan*[tiab] OR fluoroquinolone-nonsusceptible[tiab] OR fluoroquinolone-non-susceptible[tiab]))) OR ("Tuberculosis, Multidrug-Resistant"[Mesh] OR "Extensively Drug-Resistant Tuberculosis"[Mesh] OR RR-TB OR MDR-TB OR Pre-XDR-TB OR XDR-TB OR drug-resistant-TB OR multidrug-resistant-TB OR multidrug-resistant-tuberculos*[tiab] OR drug-resistant-tuberculos*[tiab] OR ((Mycobacterium-tuberculos*[tiab] OR pulmonary-tuberculos*[tiab] OR pulmonary-TB[tiab]) AND (isoniazid-resistan*[tiab] OR rifampicin-resistan*[tiab] OR floroquinolone-resistan*[tiab] OR levofloxacin-resistan*[tiab] OR moxifloxacin-resistan*[tiab] OR bedaquiline-resistan*[tiab] OR linezolid-resistan*[tiab] OR multidrug-resistan*[tiab] OR drug-resistan*[tiab]))) OR ((("Aspergillus"[Mesh] OR Aspergillus[tiab] OR a.-fumigatus[tiab] OR a.-flavus[tiab] OR Neosartorya-fumigate[tiab] OR Candida[tiab] OR c.-auris[tiab] OR c.-albicans[tiab]) AND (resistan*[tiab] OR non-susceptib*[tiab] OR nonsusceptib*[tiab])))	
**#2 -** SR	"Systematic Reviews as Topic"[Mesh] OR "Systematic Review" [Pt] OR systematic[sb] OR systematic-review*[tiab] OR meta-analysis[pt] OR meta-analysis as topic[mh] OR metaanalys*[tiab] OR meta-analys*[tiab] OR scoping[tiab]	366,960
**#3**	#1 AND #2	3,887
**#4 –** Animal Studies filter	NOT ("Animals"[Mesh] NOT ("Animals"[Mesh] AND "Humans"[Mesh]))	3,845
**#5** Specific animals	NOT (rat[ti] OR rats[ti] OR mouse[ti] OR mice[ti] OR hamster[ti] OR hamsters[ti] OR dog[ti] OR dogs[ti] OR cat[ti] OR cats[ti] OR bovine[ti] OR sheep[ti])	3,839
**#6-** Date filter	#5 AND [1-1-2012]/sd NOT [2-1-2022]/sd AND [2012-2022]/py	3,181

Web of Science
https://www.webofscience.com/

#		Results 06/12/2021
**1** – AMR	(drug-resistan* OR multidrug-resistan* OR anti-biotic-resistan* OR antibiotic-resistan* OR antibacterial-resistan* OR anti-bacterial-resistan* OR bacterial-resistan* OR bacterium-resistan* OR antimicrobial-resistan* OR microbial-resistan* OR anti-fungal-resistan* OR antifungal-resistan* OR cross-resistan* OR antibiotic-non-susceptib* OR antibiotic-nonsusceptib* OR drug-non-susceptib* OR drug-nonsusceptib*)<br>OR<br>(((Acinetobacter-baumannii* OR Achromobacter-mucosus OR Acinetobacter-baumanii OR Acinetobacter-baumanni OR Acinetobacter-genomosp.-2 OR Acinetobacter-genomospecies-2 OR Bacterium-anitratum OR Bact.-anitratum) AND (carbapenem-resistan* OR carbapenem-non-susceptib* OR carbapenem-nonsuscept*)))<br>OR<br>(((Pseudomonas-aeruginosa OR Bacillus-aeruginosus OR Bacillus-pyocyaneus OR Bacterium-aeruginosum OR Bacterium-pyocyaneum OR blue-pus-organism OR Micrococcus-pyocyaneus OR P.-aeruginosa OR Pseudomonas-polycolor OR Pseudomonas-pyoceaneus OR Pseudomonas-pyocyanea OR Pseudomonas-pyocyaneus) AND (carbapenem-resistan* OR carbapenem-non-susceptib* OR carbapenem-nonsuscept*)))<br>OR<br>(((Enterobacteriaceae OR enteric-bacteria OR enterobacteria OR enterobacteriacea OR enterobacterium OR Klebsiella-pneumonia OR Escherichia-coli OR Bacillus-coli OR Bacillus-escherichii OR Bacterium-coli OR Bacterium-coli OR bacterium-E3 OR coli-bacillus OR coli-bacterium OR colibacillus OR colon-bacillus OR E-coli OR E.-coli OR E.coli OR Enterococcus-coli OR Escherichia-alkalescens OR Enterobacter OR Serratia OR Proteus OR Providencia OR Morganella) AND (carbapenem-resistan* OR carbapenem-non-susceptib* OR carbapenem-nonsuscept* OR 3rd-generation-cephalosporin-resistan* OR third-generation-cephalosporin-resistan* OR third-generation-cephalosporin-non-susceptib*)))<br>OR<br>(VAN-R-enterococci OR VAN-R-Enterococcus OR ((Streptococcus-faecium OR Enterococcus-faecium) AND (vancomycin-resistan* OR VAN-resistan* OR vancomycin-non-susceptib* OR vancomycin-nonsusceptib*)))<br>OR<br>(MRSA OR VRSA OR ((Staphylococcus-aureus OR S.-aureus) AND (vancomycin-resistan* OR VAN-resistan* OR vancomycin-	339,733

	nonsusceptib* OR vancomycin-non-susceptib* OR Van-intermediate OR vancomycin-intermediate OR methicillin-resistan*)))<br>OR<br>(((Helicobacter-pylori OR Campylobacter-pylori* OR Helicobacter-nemestrinae) AND (clarithromycin-resistan* OR clarithromycin-non-susceptib*)))<br>OR<br>(((Campylobacter) AND (fluoroquinolone-resistan* OR fluorinated-quinolone-resistan* OR fluoro-quinolone-resistan* OR fluoroquinolones-resistan* OR fluoroquinolone-nonsusceptible OR fluoroquinolone-non-susceptible)))<br>OR<br>(((Salmonella) AND (fluoroquinolone-resistan* OR fluorinated-quinolone-resistan* OR fluoro-quinolone-resistan* OR fluoroquinolones-resistan* OR fluoroquinolone-nonsusceptible OR fluoroquinolone-non-susceptible)))<br>OR<br>(((Neisseria-gonorrhoeae OR Gonococcus-neisseri OR Gonococcus-neisseria OR Micrococcus-gonorrhoeae OR N.-gonorrhoeae OR Neisseria-gonococcus OR Neisseria-gonorrhaeae OR Neisseria-gonorrhea OR Neisseria-gonorrheae OR Neisseria-gonorrhoea) AND (3rd-generation-cephalosporin-resistan* OR third-generation-cephalosporin-resistan* OR fluoroquinolone-resistan* OR fluorinated-quinolone-resistan* OR fluoro-quinolone-resistan* OR fluoroquinolones-resistan* OR fluoroquinolone-nonsusceptible OR fluoroquinolone-non-susceptible)))<br>OR<br>(((Streptococcus-pneumoniae OR Diplococcus-pneumoniae OR Micrococcus-pneumoniae OR Pneumococcus OR pneumococcus OR Pneumococcus-pneumoniae) AND (penicillin-resistan* OR penicillin-non-susceptib* OR penicillin-nonsusceptib*)))<br>OR<br>(((Hemophilus-influenza* OR Bacterium-influenzae OR Coccobacillus-pfeifferi OR H.-influenza* OR Haemophilus-influenza* OR influenza-bacillus OR Mycobacterium-influenzae OR pfeiffer-bacillus) AND (ampicillin-resistan* OR Amp-resistan* OR ampicillin-nonsusceptib* OR ampicillin-non-susceptib*)))<br>OR<br>(((Shigella) AND (fluoroquinolone-resistan* OR fluorinated-quinolone-resistan* OR fluoro-quinolone-resistan* OR fluoroquinolones-resistan* OR fluoroquinolone-nonsusceptible OR fluoroquinolone-non-susceptible)))<br>OR<br>(RR-TB OR MDR-TB OR Pre-XDR-TB OR XDR-TB OR drug-resistant-TB OR multidrug-resistant-TB OR multidrug-resistant-tuberculos* OR drug-resistant-tuberculos* OR ((Mycobacterium-tuberculos* OR pulmonary-tuberculos* OR pulmonary-TB) AND	

	(isoniazid-resistan* OR rifampicin-resistan* OR floroquinolone-resistan* OR levofloxacin-resistan* OR moxifloxacin-resistan* OR bedaquiline-resistan* OR linezolid-resistan* OR multidrug-resistan* OR drug-resistan*)))<br>OR<br>(((Aspergillus OR a.-fumigatus OR a.-flavus OR Neosartorya-fumigate OR Candida OR c.-auris OR c.-albicans) AND (resistan* OR non-susceptib*)))	
**#2 -** SR	TS=("Systematic Review" OR (systematic NEAR/3 *review*) OR metaanalys* OR meta-analys* OR met*analys* OR (scoping NEAR/3 (literature OR *review*)))	602,250
**#3**	#1 AND #2	5,279
**#4** Specific animals	NOT TI=(rat OR rats OR mouse OR mice OR hamster OR hamsters OR dog OR dogs OR cat OR cats OR bovine OR sheep)	5,254
**#5-** Date filter	#5 AND Timespan: 2012-01-01 to 2022-01-01 (Index Date)	4,428

Supplementary Table 2. Data attributes to be extracted

**Document-level attributes**	
Author(s);	
Author type	- Government agency;<br>- International (technical) agency/organization;<br>- Funding agency/donor;<br>- Research organization/university;<br>- Expert group/professional association;<br>- Individual authors;<br>- Audit/accreditation body;<br>- Private for-profit organization;<br>- Non-government organization;<br>- Other<br>- Unassigned
Journal/Book	Open text
Publisher	Open text
Publication year	
Country/countries of origin	Open text
Funding source	Open text
Information source	- WHO document;<br>- WHO guideline;<br>- Grey literature;<br>- Systematic reviews in the bibliographic database;<br>- Scientific papers suggested by the WHO AMR Steering Group
Purpose/Type of document	- Guidance/guideline;<br>- Report;<br>- Systematic review/meta-analysis;<br>- Narrative review/commentary/editorial;<br>- Other
Data extractor 1 and 2	
Date of data extraction 1 and 2	
Confirmation on document's inclusion/exclusion criteria	Document inclusion criteria:<br>1. Document describes one or more knowledge gaps or research questions on AMR, including priorities, framework, components, elements, or steps for the description (i.e., epidemiology, burden and drivers), delivery, development and/or discovery of tools, products, or interventions for AMR prevention, diagnosis and/or care & treatment.<br>2. Document has a global or regional application and/or relevance for LMIC.<br>Document exclusion criteria:

	1. Document only describes individual research studies or case reports;<br>2. Document is not related to bacteria included in the WHO priority pathogen list, M. tuberculosis or critical fungi (e.g., parasites, viruses);<br>3. Document is related to AMR in the non-human sectors;<br>4. Document has no identifiable authors, publisher and year of publication.
**Document-level and research-question level attributes**	
Geographical scope	- Unassigned<br>- Global<br>- Regional;<br>- National;<br>- Subnational/local
Region (if applicable)	Open text;
Country/-ies (if applicable)	Open text;
Resource/socioeconomic context (World Bank definition)	- Unassigned<br>- General/overall<br>- LIC<br>- MIC<br>- HIC<br>- Low- and Middle-Income Countries (LMIC)
Population	- Unassigned<br>- General/overall<br>- Adult<br>- Children<br>- Neonates<br>- Elderly<br>- Immunocompromised<br>- Comorbidities<br>- Pregnant women<br>- Other vulnerable groups
Setting	- Unassigned<br>- General/overall<br>- Community<br>- Health care system<br>o Primary health care<br>o Acute-care hospitals (including secondary and tertiary care)<br>o Long-term care facilities (including nursing homes)<br>o ICU
Micro-organism group	- Unassigned<br>- General/overall<br>- PPL<br>- TB

	- Critical fungi
Antibiotic-microorganism combination (refer to Table 1)<br>-	- Unassigned<br>- General/overall<br>- Acinetobacter baumannii: carbapenem-resistant<br>- Pseudomonas aeruginosa: carbapenem-resistant<br>- Enterobacteriaceae (including Klebsiella pneumonia, Escherichia coli, Enterobacter spp., Serratia spp., Proteus spp., and Providencia spp, Morganella spp.): carbapenem-resistant, 3rd generation cephalosporin-resistant<br>- Enterococcus faecium: vancomycin-resistant<br>- Staphylococcus aureus: methicillin-resistant, vancomycin-intermediate and -resistant<br>- Helicobacter pylori: clarithromycin-resistant<br>- Campylobacter: fluoroquinolone-resistant<br>- Salmonella spp.: fluoroquinolone-resistant<br>- Neisseria gonorrhoeae: 3rd generation cephalosporin-resistant, fluoroquinolone-resistant<br>- Streptococcus pneumoniae: penicillin-non-susceptible<br>- Haemophilus influenzae: ampicillin-resistant<br>- Shigella spp.: fluoroquinolone-resistant<br>- RR-TB<br>- MDR-TB<br>- Pre-XDR-TB<br>- XDR-TB<br>- Aspergillus spp (including fumigatus and flavus)<br>- Candida spp. (including albicans and auris).
Syndrome	- Unassigned<br>- General/overall<br>- Bloodstream infection/sepsis<br>- Pneumonia/respiratory tract<br>- Gastrointestinal infection<br>- Urinary tract infection<br>- Skin and soft tissue<br>- Central nervous system infection<br>- Sexually Transmitted Infection<br>- TB-general/unspecified<br>- TB-pulmonary<br>- TB-extrapulmonary<br>- Other
Sector	- Human health sector<br>- One health (intersection/overlapping between animal, environment and human health sector)
Topic	- Unassigned<br>- General/overall<br>- AMR National Action Plans and Monitoring & Evaluation<br>- AMR surveillance<br>- Antimicrobial consumption surveillance

	- AMR One Health<br>- Antimicrobial Use/AMR Stewardship/Essential Medicines<br>- Antimicrobial R&D<br>- AMR awareness<br>- Diagnostics (including R&D)<br>- Infection Prevention and Control<br>- Water, Sanitation, Hygiene and Health<br>- Immunization/Vaccine (including Product & Delivery Research)<br>- Food safety/food borne diseases<br>- Sexually Transmitted Infections<br>- TB Prevention, Diagnosis, Treatment, Care & Innovation<br>- Newborn health<br>- Health systems strengthening/Universal Health Coverage/Policy and regulatory
**Research-question level attributes**	
Theme	- Prevention (subcategories for CAI and HAI, immunization, IPC, other)<br>- Diagnosis<br>- Care & treatment (subcategories: CAI and HAI)<br>- Unassigned
Domain	- Descriptive (subcategories: measuring the burden; understanding risk factors; measuring prevalence of exposure to risk factors; Evaluating the efficacy and effectiveness of interventions in place; measuring prevalence of coverage of interventions in place)<br>- Delivery (subcategories: Health policy analysis; Health system structure analysis; Financing/costs analysis; Human resources; Provision/infrastructure; Operations research; Responsiveness/recipients)<br>- Development (subcategories: acceptability, affordability, deliverability/feasibility, sustainability)<br>- Discovery (subcategories: medicines, diagnostics, vaccines, digital health technology, other preventive tools, other)<br>- Unassigned
Area	- Epidemiology (including drivers, prevalence and burden)<br>- Pharmacological and clinical<br>- Behavioural and social science<br>- Health economics/cost-effectiveness<br>- Legislation and regulation

Abbreviations: AMR: antimicrobial resistance; CAI: community-acquired infection; HAI: healthcare-associated infection; HIC: high-income country; ICU: intensive care unit; IPC: infection prevention and control; LIC: low-income country; LMIC: low- and middle-income country; MIC: middle-income country MDR-TB: multidrug-resistant tuberculosis; PPL: WHO priority pathogen list; Pre-XDR-TB: pre-extensively drug-resistant tuberculosis; RR-TB:

rifampicin-resistant tuberculosis; R&D: research and development; TB: tuberculosis; XDR-TB: extensively drug-resistant tuberculosis
