# Supplementary material for: Developing a priority global research agenda for antimicrobial resistance in the human health sector: protocol for a scoping review
Source: BMJ Open. 2022 Jun 2;12(6):e060553. doi: 10.1136/bmjopen-2021-060553 (PMC9163534; doi:10.1136/bmjopen-2021-060553)
Supplement: Author's manuscript [file bmjopen-2021-060553.draft_revisions.pdf]

BMJ Open

BMJ Open is committed to open peer review. As part of this commitment we make the peer review history of every article we publish publicly available.

When an article is published we post the peer reviewers' comments and the authors' responses online. We also post the versions of the paper that were used during peer review. These are the versions that the peer review comments apply to.

The versions of the paper that follow are the versions that were submitted during the peer review process. They are not the versions of record or the final published versions. They should not be cited or distributed as the published version of this manuscript.

BMJ Open is an open access journal and the full, final, typeset and author-corrected version of record of the manuscript is available on our site with no access controls, subscription charges or pay-per-view fees (http://bmjopen.bmj.com).

If you have any questions on BMJ Open's open peer review process please email info.bmjopen@bmj.com

BMJ Open

Developing a priority global research agenda for antimicrobial resistance in the human health sector: protocol for a scoping review

Journal:	*BMJ Open*
Manuscript ID	bmjopen-2021-060553
Article Type:	Protocol
Date Submitted by the Author:	28-Dec-2021
Complete List of Authors:	Hamers, Raph Leonardus; University of Oxford Medical Sciences Division, Eijkman-Oxford Clinical Research Unit; University of Oxford Medical Sciences Division, Nuffield Department of Medicine<br>Cassini, Alessandro; World Health Organization, Department of Control, Surveillance and Prevention of Antimicrobial Resistance<br>Asadinia, Koe Stella; University of Oxford Medical Sciences Division, Eijkman-Oxford Clinical Research Unit<br>Bertagnolio, S; World Health Organization, Department of Control, Surveillance and Prevention of Antimicrobial Resistance
Keywords:	INFECTIOUS DISEASES, Diagnostic microbiology < INFECTIOUS DISEASES, Epidemiology < INFECTIOUS DISEASES, Infection control < INFECTIOUS DISEASES, Public health < INFECTIOUS DISEASES

SCHOLARONE™
Manuscripts

Developing a priority global research agenda for antimicrobial resistance in the human health sector: protocol for a scoping review

Authors

Raph L. Hamers[1,2], Alessandro Cassini[3], Koe Stella Asadinia[1], Silvia Bertagnolio[3]

Affiliations

1. Eijkman-Oxford Clinical Research Unit, Jakarta, Indonesia;
2. Centre for Tropical Medicine and Global Health, Nuffield Department of Medicine, University of Oxford, Oxford, UK;
3. Control and Response Strategies (CRS) Unit, Department of Control, Surveillance and Prevention of Antimicrobial Resistance, World Health Organization, Geneva, Switzerland

Correspondence to

Silvia Bertagnolio, MD

Control and Response Strategies (CRS) Unit

Department of Control, Surveillance and Prevention of Antimicrobial Resistance

World Health Organization

20, Avenue Appia

1211 Geneva 27, Switzerland

Mobile: +41 79 7288078

Office +41 22 791 3958

Email: bertagnolios@who.int

Word count: 2505

Inserts Tables 3; Panel 1

Supplemental material: Tables 2

For peer review only

Abstract (323w)

Introduction To accelerate the response to the public health threat by antimicrobial resistance (AMR), WHO is developing a Global Research Agenda for AMR in the human health sector that aims to provide a global and transparent assessment of priority knowledge gaps that inform control and response strategies to tackle AMR. The Agenda identifies gaps related to critical bacteria and fungi and Mycobacterium tuberculosis to be addressed by 2030. A literature scoping review represents the first phase in a stepwise process, and we hereby outline the protocol to review research questions related to AMR in the human health sector.

Methods and analysis This scoping review will follow the Arksey and O'Malley (2005) methodology. A hand search will be conducted to identify relevant WHO guidelines and other documents. The grey literature will be searched through a stakeholder mapping process and google searches of organisational websites. Bibliographic databases (PubMed, Embase and Web of Science) will be systematically searched for relevant systematic reviews. We will also screen documents suggested by the WHO Steering Group for the AMR Global Research Agenda as well as the reference lists of included studies. Two researchers separately will review the yielded citations to determine eligibility based on pre-defined criteria. Relevant research questions with attributes will be extracted using a tool developed through an iterative process by the research team. Each identified research question will be classified and aggregated according to a conceptual framework (i.e. "knowledge matrix"), composed of three themes aligned with the WHO AMR patient pathway framework (i.e. Prevention, Diagnosis and Care & Treatment) and four cross-cutting domains (i.e. Descriptive, Discovery, Development, Delivery). We will present numerical and thematic summaries of the knowledge matrix, and will not conduct a qualitative content analysis.

Ethics and dissemination The findings will be disseminated through a peer-reviewed publication and stakeholder meetings.

For peer review only - http://bmjopen.bmj.com/site/about/guidelines.xhtml

Keywords antimicrobial resistance; knowledge gaps, research gaps, research questions

Article Summary: Strengths and limitations of this study

- This is a novel scoping review that aims to identify research questions and knowledge gaps on AMR, as the first phase in a stepwise process for developing a priority AMR Global Research Agenda, in consultation with the WHO Steering Group for the AMR Global Research Agenda, external experts and stakeholders.
- Adopting the modified standardised method for setting priorities in health research developed by the Child Health and Nutrition Research Initiative (CHNRI), the scoping review will populate a pre-defined conceptual framework to capture and categorize information on the description, delivery, development and/or discovery of interventions for AMR prevention, diagnosis and/or care & treatment ("knowledge matrix").
- The identification and synthesis of data will be limited to WHO documents, grey literature and systematic reviews published in the past 10 years, to capture the most important research questions and knowledge gaps and inform the further consultation process.

For peer review only - http://bmjopen.bmj.com/site/about/guidelines.xhtml

INTRODUCTION

Background

The global rise in antimicrobial resistance (AMR) is considered one of the greatest public health threats, with a growing and disproportionate impact in low- and middle-income countries (LMIC).[1] One of the major drivers of AMR is antimicrobial use, especially their misuse and overuse, in humans and animals for human consumption.[2] Lack of access to clean water and sanitation in low-resource settings and inadequate infection prevention and control promotes the spread of microbes, including those resistant to antimicrobial treatment. Access to life-saving drugs targeting the emerging resistant pathogens remains an issue in LMIC, while much has been written about the dire state of the pipeline of new antibacterial drugs. These realities occur in a wider context of lack of access to quality health care in many settings. Key strategies to address AMR are to reduce the need for antibiotics (through prevention of infections), to reduce the use of antibiotics (by better use of diagnostics and antibiotic stewardship), and to find new methods of disease prevention and treatment.

To address the serious public health threat posed by AMR, WHO coordinated the development of a Global Action Plan (GAP) on AMR in 2015, which in turn has been translated into National Action Plans.[3] To accelerate their implementation, there is a need to expand the evidence-base on AMR burdens, impact and interventions, including their prioritization, cost-effectiveness, financing and knowledge on how to bring existing interventions at scale.[4–6] Since their implementation has been further set back because of the COVID-19 pandemic, now more than ever, there is an urgent need to revitalize work on AMR.[7] Appropriate targeted research is needed to make substantial progress in attaining the 2030 Sustainable Development Goals

For peer review only - http://bmjopen.bmj.com/site/about/guidelines.xhtml

(SDGs) and to provide the evidence-base in support of successful implementation of interventions to prevent, diagnose and manage drug-resistant infections.

The WHO is developing a priority Global Research Agenda for AMR that aims to provide a global and transparent assessment of knowledge gaps in the human health sector that –once translated into evidence– inform control and response strategies to tackle AMR in the human health sector, catalyse investment and scientific interest among researchers, donors and public health professionals towards the generation of new evidence to address knowledge gaps for effective implementation of global, regional and national policies on AMR, particularly LMIC, in the timeframe 2023-2030. The evidence generated will inform prioritization of interventions within AMR National Action Plans. This work focuses on the human health sector only, and will complement the WHO One Health Priority Research Agenda for AMR, being developed in parallel, which focuses on the interface between humans, animals, plants and their shared environment.

Aim and Objectives

The first phase of the AMR Global Research Agenda project is to conduct a scoping literature review to identify existing research questions and knowledge gaps on AMR on:

- Burden of AMR and what are the factors associated with and predictive of AMR emergence and transmission in the human health sector
- New or improved interventions, technologies and tools that are associated with improved AMR prevention, diagnosis, care and treatment and best ways to deliver these

For peer review only

METHODS AND ANALYSIS

Conceptual framework and scope

The development of the AMR Global Research Agenda will follow the WHO methodological framework *Guidance for undertaking a research priority setting exercise*,[8] which uses a modified version of the standardised method for setting priorities in health research developed by the Child Health and Nutrition Research Initiative (CHNRI).[9,10] The CHNRI framework proposes a flexible, systematic approach to listing a large number of research options and research questions, and allows for a prioritisation exercise that is systematic, transparent, legitimate and fair, scientifically rigorous and replicable. Evidence needed should be clearly framed by the research options or questions, and the effect of its translation and implementation into interventions should be straightforward. The CHNRI framework uses the “4D framework” of “description”, “delivery”, “development” and “discovery” research. [9,10] Each identified research question will thus be classified and aggregated to populate a “knowledge matrix”, composed of three focus areas or "themes" broadly aligned with the WHO AMR patient pathway framework (i.e., Prevention, Diagnosis and Care & Treatment) and the four cross-cutting domains (Descriptive, Discovery, Development, Delivery), as described in **Table 1**.

Table 1. Knowledge matrix

		**Themes (within the AMR patient pathway)**		
		**Prevention**	**Diagnosis**	**Care & Treatment**
**Cross-cutting domains (“4Ds”)**	**Descriptive**			
	**Delivery**			
	**Development**			
	**Discovery**			

For peer review only - http://bmjopen.bmj.com/site/about/guidelines.xhtml

Table footnote: Definitions	
Theme/Domain	Description
Prevention	Prevention of AMR in hospital- and community-acquired infections
Diagnosis	Diagnosis of infections caused by microorganisms resistant to antimicrobials, including both pathogen and AMR identification
Care & treatment	Care and treatment of hospital- and community-acquired infections caused by antimicrobial-resistant micro-organisms
Descriptive	Improve understanding of AMR epidemiology, burden and factors associated with and predictive of AMR emergence and transmission (epidemiological research)
Delivery of existing interventions	Optimize and improve the delivery of existing interventions (implementation research, operations research, and/or health policy and systems research)
Development of improved interventions	Improve existing interventions by reducing costs, or optimizing uptake, impact, sustainability and feasibility
Discovery of new interventions	New tools and interventions (new medicines, diagnostics, vaccines, or other interventions)

Abbreviations: AMR: antimicrobial resistance

To populate the “knowledge matrix” as a first step in this iterative process, we will conduct a scoping review of existing research questions through reviews of published and grey literature. The scoping review will also be informed by experts in the WHO Steering Group for the AMR Global Research Agenda (SG) composed of

For peer review only

technical experts in areas relevant to AMR, including AMR National Action Plans and Monitoring & Evaluation; AMR surveillance and antimicrobial use; AMR stewardship and awareness; antimicrobial R&D; WHO essential medicines; diagnostics; infection prevention and control (IPC); water, sanitation, hygiene and health (WASH); vaccine/product & delivery research; the Special Programme for Research and Training In Tropical Diseases (TDR); food safety/foodborne diseases; sexually transmitted infections; newborn health; and tuberculosis (TB) prevention, diagnosis, treatment, care & innovation. The scope of the scoping review is summarized in **Panel**.

Panel: Scope of the scoping review

The scoping review:

- Is global in its scope;

- Focuses exclusively on AMR in the human health sector;

- Focuses on priority research questions that can and should be addressed in the timeframe 2023-2030 (SDGs)

- Is based on a “knowledge matrix” composed of three focus areas or "themes" (broadly aligned with the WHO AMR patient pathway framework: Prevention, Diagnosis and Care & Treatment) and four cross-cutting domains (Descriptive, Discovery, Development, Delivery)

- The following disaggregation is considered when categorizing the research questions:

    - Populations (e.g., adults, children, neonates, vulnerable groups);
    - Settings (e.g., community, primary care, hospitals);
    - Geographical/socioeconomic context (e.g., LMIC, HIC)
    - Syndrome (e.g., bloodstream infections, etc)
    - Antibiotic-microorganism (“drug-bug”) combinations

- Will not consider prioritization of specific pathogens;

- Focuses on the WHO Global priority list of antibiotic-resistant pathogens [11]:

For peer review only - http://bmjopen.bmj.com/site/about/guidelines.xhtml

- *Acinetobacter baumannii*: carbapenem-resistant
- *Pseudomonas aeruginosa*: carbapenem-resistant
- *Enterobacteriaceae* (including *Klebsiella pneumonia, Escherichia coli, Enterobacter spp., Serratia spp., Proteus spp.,* and *Providencia spp, Morganella spp.*): carbapenem-resistant, 3rd generation cephalosporin-resistant
- *Enterococcus faecium*: vancomycin-resistant
- *Staphylococcus aureus*: methicillin-resistant, vancomycin-intermediate and -resistant
- *Helicobacter pylori*: clarithromycin-resistant
- *Campylobacter*: fluoroquinolone-resistant
- *Salmonella spp.*: fluoroquinolone-resistant
- *Neisseria gonorrhoeae*: 3rd generation cephalosporin-resistant, fluoroquinolone-resistant
- *Streptococcus pneumoniae*: penicillin-non-susceptible
- *Haemophilus influenzae*: ampicillin-resistant
- *Shigella spp.*: fluoroquinolone-resistant

- Focuses on critical fungi, i.e.:

- *Aspergillus spp.* (especially *A. fumigatus* and *A. flavus*)
- *Candida spp.* (especially *C. albicans* and *C. auris*).

- Focuses on drug-resistant *Mycobacterium tuberculosis*, with the following definitions:

- RR-TB: rifampicin-resistant TB;
- MDR-TB: resistance to at least isoniazid and rifampicin;
- Pre-XDR-TB: MDR/RR-TB and also resistant to any fluoroquinolone.
- XDR-TB: pre-XDR-TB and also resistant to at least one additional Group A drug (comprises levofloxacin, moxifloxacin, bedaquiline and linezolid).

Abbreviations: AMR, antimicrobial resistance; HIC: high-income country; LMIC, low- and middle-income countries; MDR-TB, multidrug-resistant tuberculosis; Pre-XDR-TB, pre-extensively drug-resistant tuberculosis; RR-TB, rifampicin-resistant

For peer review only - http://bmjopen.bmj.com/site/about/guidelines.xhtml

For peer review only

tuberculosis; SDGs, sustainable development goals; XDR-TB, extensively drug-resistant tuberculosis.

Protocol design

This protocol follows the methodological framework for scoping reviews developed by Arksey and O'Malley and Levac *et al*. through six stages, as well as the guidance developed by the Joanna Briggs Institute to standardize the conduct of scoping reviews.[12] Due to the iterative nature of a scoping review, protocol deviations during the process may be necessary. Any discrepancies will be detailed and justified in the Methods section of the final report.

Stage 1: Identifying the research question

The purpose of the scoping review is to map the literature and identify the key research questions and knowledge gaps that inform the research priority setting exercise to develop the AMR Global Research Agenda. This scoping review addresses the following broad question: "What are the existing research questions and knowledge gaps on the description, delivery, development and/or discovery of interventions for AMR prevention, diagnosis and/or care & treatment in the human health sector, at a global level?"

Stage 2: Searching for relevant documents

We plan to search several information sources of published and grey literature to identify relevant documents as listed in **Table 3**.

Table 3. List of information sources to be searched for the scoping review

1. Previous and current WHO-authored documents and research gaps identified through WHO guidelines processes in areas relevant to AMR

For peer review only - http://bmjopen.bmj.com/site/about/guidelines.xhtml

• Documents searched include strategies, global action plans, meeting reports, policy briefs, technical documents, resolutions.<br>• We will manually search the WHO Institutional Repository for Data Sharing (IRIS), Data Platform, The Global Health Observatory, and WHO Observatory for Health R&D.
2. Grey literature relevant to AMR
• This includes position papers, reports, strategies, guidelines, and evaluations from national or international agencies and AMR stakeholders.<br>• We will first conduct a global mapping of stakeholders working with AMR policy, advocacy, innovation and research, surveillance, based on the ReAct paper published on this topic,[13] organisational websites, and feedback from the WHO Steering Group for the AMR Global Research Agenda, followed by a targeted manual search of organisational websites.<br>• This will be supplemented with a google search including the term “filetype:pdf” and “antimicrobial resistance” (and synonyms).
3. Published systematic reviews on AMR research gaps and priorities
• A systematic search of the relevant bibliographic databases, i.e., PubMed, Embase and Web of Science (Full search terms in **Supplementary Table 1**)
4. Published scientific papers, editorials, reviews and grey literature suggested by **WHO Steering Group for the AMR Global Research Agenda**

Abbreviations: AMR, antimicrobial resistance; R&D, research and development

All searches will be jointly developed and conducted by an experienced WHO librarian, the authors and content experts in the SG. We will manually search the grey literature in organisations' websites and google, and consider any relevant documents as suggested by the SG. The full search terms for the bibliographic databases are available in **Supplementary Table 1.** For the non-general data sources, we will use less restrictive terms to be able to identify relevant documents.

For peer review only - http://bmjopen.bmj.com/site/about/guidelines.xhtml

We will search reference lists of included documents for any additional potentially relevant references. The searches will be restricted to the past 10 years and the English language. After the searches, the SG will review the search results to determine if the documents identified represent all relevant resources.

Stage 3: Document selection

This stage will be an iterative process involving searching the literature, refining the search strategy, and reviewing articles for eligibility and inclusion. The study team will meet to discuss study inclusion and exclusion criteria at the beginning of the process, and will discuss challenges and uncertainties related to study selection along the review process. The study team will go back and refine the search strategy if needed. For grey literature, a first reviewer will screen all full-text documents for eligibility, and a second reviewer will dual-screen all full-text documents. For bibliographic databases, a first reviewer will screen all titles and abstracts for eligibility, and a second reviewer will screen at least 20% of included and excluded titles and abstracts; if the agreement is <80%, a second reviewer will dual-screen all titles and abstracts. Then, a single reviewer will read all included full-text articles, and a second reviewer will read 20% of included and excluded full-text articles; if the agreement is <80%, a second reviewer will dual read all included full-text articles. We will resolve disagreements on study selection by consensus and discussion with other reviewers as needed. For grey literature, duplicate documents will be removed in Mendeley manually and using the automated tool. For bibliographic databases, duplicate documents will be removed in EndNote using the Bramer method.[14] Documents that are superseded with a more recent version are excluded (and the most recent is included).

Inclusion criteria:

For peer review only - http://bmjopen.bmj.com/site/about/guidelines.xhtml

1. Document describes one or more knowledge gaps or research questions on AMR, including priorities, framework, components, elements, or steps for the description (i.e., epidemiology, burden and drivers), delivery, development and/or discovery of tools, products, or interventions for AMR prevention, diagnosis and/or care & treatment.
2. Document has a global or regional application and/or relevance for LMIC.

Exclusion criteria:

1. Document only describes individual research studies or case reports;
2. Related to microorganisms not included in the WHO priority pathogen list, TB or critical fungi (e.g., parasites, viruses);
3. Non-human sectors;
4. Document has no identifiable authors, publisher and year of publication.

For peer review only

Stage 4: Extracting the data

Data elements will be extracted from included documents using a data extraction form in Microsoft Excel, which will be jointly developed by the research team and updated during the iterative extraction process ("charting the data"). Two reviewers will independently test the data extraction form on 5-10 documents to ensure all relevant results are extracted and that their approach to extraction is consistent with the research question and purpose. The content of the included documents will be read in-depth to identify research questions and knowledge gaps within the scope of the scoping review. A single reviewer will extract the data, and translate each research gap, need or question into one or more research questions that are specific for the attributes expressed in the source text. The extracted data and research questions will be reviewed and refined by at least two other team members. The research team will meet regularly to review the research questions identified and ensure data extraction is consistent with the research question and purpose.

For peer review only - http://bmjopen.bmj.com/site/about/guidelines.xhtml

Document authors will not be contacted. We will use a "narrative review" or "descriptive analytical" method to extract information from each document. **Supplementary Table 2** specifies the data attributes that will be extracted.

Stage 5: Collating, summarizing and reporting results

We will present a numerical analysis of the characteristics of included documents (using tables and charts), and we will use the thematic construction of the "knowledge framework" (**Table 2**) to provide an overview of the breadth of the literature (i.e., descriptive numerical and thematic summaries). We will report the results and outcomes and consider the meaning of the findings as they relate to the overall study purpose. However, we will not conduct a qualitative content analysis or synthesis, nor will we -at this stage- discuss implications for future research, practice and policy. The findings of the scoping review will form a basis for the next steps in developing the WHO Global Research Agenda for AMR. We will not perform a formal assessment of the methodological quality of the included documents as the purpose of this scoping review is to achieve a broad and in-depth description of existing research questions and knowledge gaps around AMR. We will report in compliance with the PRISMA extension for Scoping Reviews (PRISMA-ScR),[15] including a flow diagram for the scoping review process and a completed checklist.

Stage 6: Consulting with stakeholders

The scoping review process will be conducted in regular consultation with the SG to inform and validate the results. Later in the agenda-setting process, various rounds of expert and stakeholder consultations will be conducted.

Patient and public involvement statement

The patient and public were not involved in the development of this study protocol.

For peer review only - http://bmjopen.bmj.com/site/about/guidelines.xhtml

ETHICS AND DISSEMINATION

The scoping review process will only involve identification, selection and analysis of documents available for use in the public domain, therefore ethical approval is not required. The scoping review will be done in consultation with the SG and disseminated through a peer-reviewed publication. This scoping review is the first step in a stepwise process for developing a priority AMR Global Research Agenda, which will be done in consultation with global, regional and national stakeholders who are the end-users of the generated knowledge and disseminated through a final report.

For peer review only

Authors' contributions

AC and SB conceptualised the project. RLH, AC and SB developed the protocol and search strategies. RLH drafted the manuscript, with critical input from AC, KSA and SB. KSA designed and tested the data extraction tools. All authors critically reviewed the paper for important intellectual content and approved the final version of the manuscript.

Acknowledgements

The authors would like to acknowledge the contributions and suggestions of the WHO Steering Group for the AMR Global Research Agenda in the Human Health Sector: Kitty Van Weezenbeek, Sarah Paulin, Berbara Tornimbene, Tine Jorgensen, Peter Beyer, Thomas Joseph, Benedetta Allegranzi, Nebiat Gebreselassie, Mateusz Hasso-Agopsowicz, Rob Terry, Benedikt Huttner, Bruce Gordon, Francis Moussy, Luz De Regil, Sachiyo Yoshida, Teodora Wi. The authors also acknowledge the contributions and suggestions of other WHO colleagues: Tomas Allen, Anand Balachandran, Verica Ivanovska, Kavita Kothari, Kate Medlicott, Arno Muller, Maarten van der Heijden and Matteo Zignol.

Funding statement

This work is part of the project entitled “Global Research Agenda for Antimicrobial Resistance in the Human Health Sector” initiated and funded by WHO.

Competing interests statement

AC and SB are WHO employees. RLH is a consultant for this work and is also supported by the Wellcome Trust (106680/Z/14/Z). The views expressed are those of the authors and not necessarily those of WHO, Wellcome Trust, or any of the institutions mentioned.

For peer review only

Data sharing statement: No additional data are available.

ORCID IDs:

Raph L. Hamers 0000-0002-5007-7896

Alessandro Cassini 0000-0002-7973-6445

Koe Stella Asadinia 0000-0003-4552-5014

Silvia Bertagnolio 0000-0001-8416-2581

For peer review only

References

1 Sriram A, Kalanxhi E, Kapoor G, *et al.* Center for Disease Dynamics, Economics & Policy, Washington DC. State of the world's antibiotics 2021: A global analysis of antimicrobial resistance and its drivers. 2021.

2 Klein EY, Van Boeckel TP, Martinez EM, *et al.* Global increase and geographic convergence in antibiotic consumption between 2000 and 2015. *Proc Natl Acad Sci* 2018;:201717295. doi:10.1073/pnas.1717295115

3 World Health Organisation. Global action plan on antimicrobial resistance. 2015. https://www.who.int/publications/i/item/9789241509763

4 Laxminarayan R, Van Boeckel T, Frost I, *et al.* The Lancet Infectious Diseases Commission on antimicrobial resistance: 6 years later. *Lancet Infect Dis* 2020;**20**:e51–60. doi:10.1016/S1473-3099(20)30003-7

5 Laxminarayan R, Duse A, Wattal C, *et al.* Antibiotic resistance-the need for global solutions. Lancet Infect. Dis. 2013;**13**:1057–98. doi:10.1016/S1473-3099(13)70318-9

6 Wernli D, Jørgensen PS, Parmley EJ, *et al.* Evidence for action: a One Health learning platform on interventions to tackle antimicrobial resistance. *Lancet Infect Dis* 2020;**20**:e307–11. doi:10.1016/S1473-3099(20)30392-3

7 Tomczyk S, Taylor A, Brown A, *et al.* Impact of the COVID-19 pandemic on the surveillance, prevention and control of antimicrobial resistance: a global survey. *J Antimicrob Chemother* Published Online First: 2 September 2021. doi:10.1093/jac/dkab300

8 Organization WH. *A systematic approach for undertaking a research priority-setting exercise: guidance for WHO staff*. Geneva PP - Geneva: : World Health Organization https://apps.who.int/iris/handle/10665/334408

9 Rudan I, Gibson JL, Ameratunga S, *et al.* Setting priorities in global child health research investments: guidelines for implementation of CHNRI method.

For peer review only

For peer review only - http://bmjopen.bmj.com/site/about/guidelines.xhtml

Croat Med J 2008;**49**:720–33. doi:10.3325/cmj.2008.49.720

10 Rudan I. Setting health research priorities using the CHNRI method: IV. Key conceptual advances. *J Glob Health* 2016;**6**:10501. doi:10.7189/jogh-06-010501

11 World Health Organization. Prioritization of pathogens to guide discovery, research and development of new antibiotics for drug-resistant bacterial infections, including tuberculosis. 2017.

12 Peters MDJ, Godfrey CM, Khalil H, *et al.* Guidance for conducting systematic scoping reviews. *JBI Evid Implement* 2015;**13**.https://journals.lww.com/ijebh/Fulltext/2015/09000/Guidance_for_conducting_systematic_scoping_reviews.5.aspx

13 ReAct. AMR stakheolder mapping. A global mapping of stakeholders working with antimicrobial resistance. 2016.

14 Bramer WM, Giustini D, de Jonge GB, *et al.* De-duplication of database search results for systematic reviews in EndNote. *J Med Libr Assoc* 2016;**104**:240–3. doi:10.3163/1536-5050.104.3.014

15 Tricco AC, Lillie E, Zarin W, *et al.* PRISMA Extension for Scoping Reviews (PRISMA-ScR): Checklist and Explanation. *Ann Intern Med* 2018;**169**:467–73. doi:10.7326/M18-0850

For peer review only

Supplemental material

Supplementary Table 1. Full search terms for the bibliographic databases

Embase
http://www.embase.com

#		Results 10/12/2021
**1** – AMR	('drug resistance'/de OR 'antibiotic resistance'/exp OR 'antifungal resistance'/exp OR 'cross resistance'/exp OR 'extensive drug resistance'/exp OR 'multidrug resistance'/exp OR (drug-resistan* OR multidrug-resistan* OR anti-biotic-resistan* OR antibiotic-resistan* OR antibacterial-resistan* OR anti-bacterial-resistan* OR bacterial-resistan* OR bacterium-resistan* OR antimicrobial-resistan* OR microbial-resistan* OR anti-fungal-resistan* OR antifungal-resistan* OR cross-resistan* OR antibiotic-non-susceptib* OR antibiotic-nonsusceptib* OR drug-non-susceptib* OR drug-nonsusceptib*):ab,ti)<br>OR<br>('carbapenem resistant Acinetobacter baumannii'/exp OR ((Acinetobacter-baumannii* OR Achromobacter-mucosus OR Acinetobacter-baumanii OR Acinetobacter-baumanni OR Acinetobacter-genomosp.-2 OR Acinetobacter-genomospecies-2 OR Bacterium-anitratum OR Bact.-anitratum) AND (carbapenem-resistan* OR carbapenem-non-susceptib* OR carbapenem-nonsuscept*)):ti,ab)<br>OR<br>('carbapenem resistant Pseudomonas aeruginosa'/exp OR ((Pseudomonas-aeruginosa OR Bacillus-aeruginosus OR Bacillus-pyocyaneus OR Bacterium-aeruginosum OR Bacterium-pyocyaneum OR blue-pus-organism OR Micrococcus-pyocyaneus OR P.-aeruginosa OR Pseudomonas-polycolor OR Pseudomonas-pyoceaneus OR Pseudomonas-pyocyanea OR Pseudomonas-pyocyaneus) AND (carbapenem-resistan* OR carbapenem-non-susceptib* OR carbapenem-nonsuscept*)):ti,ab)<br>OR<br>('carbapenem-resistant Enterobacteriaceae'/exp OR ((Enterobacteriaceae OR enteric-bacteria OR enterobacteria OR enterobacteriacea OR enterobacterium OR Klebsiella-pneumonia OR Escherichia-coli OR Bacillus-coli OR Bacillus-escherichii OR Bacterium-coli OR Bacterium-coli OR bacterium-E3 OR coli-bacillus OR coli-bacterium OR colibacillus OR colon-bacillus OR E-coli OR E.-coli OR E.coli OR Enterococcus-coli OR Escherichia-alkalescens OR Enterobacter OR Serratia OR Proteus OR Providencia OR Morganella) AND (carbapenem-resistan* OR carbapenem-non-susceptib* OR carbapenem-nonsuscept* OR 3rd-generation-cephalosporin-resistan* OR third-generation-cephalosporin-resistan* OR third-generation-cephalosporin-non-susceptib*)):ti,ab)<br>OR	513,168

	('vancomycin resistant Enterococcus'/exp OR (VAN-R-enterococci OR VAN-R-Enterococcus OR (Streptococcus-faecium OR Enterococcus-faecium) AND (vancomycin-resistan* OR VAN-resistan* OR vancomycin-non-susceptib* OR vancomycin-nonsusceptib*)):ti,ab)<br>OR<br>('methicillin resistant Staphylococcus aureus'/exp OR 'methicillin resistant Staphylococcus aureus infection'/exp OR 'vancomycin resistant Staphylococcus aureus'/exp OR 'vancomycin intermediate Staphylococcus aureus'/exp OR (MRSA OR VRSA OR (Staphylococcus-aureus OR S.-aureus) AND (vancomycin-resistan* OR VAN-resistan* OR vancomycin-nonsusceptib* OR vancomycin-non-susceptib* OR Van-intermediate-resistan* OR vancomycin-intermediate-resistan* OR methicillin-resistan* OR methicillin-nonsusceptib* OR methicillin-non-susceptib*)):ti,ab)<br>OR<br>(((Helicobacter-pylori OR Campylobacter-pylori* OR Helicobacter-nemestrinae) AND (clarithromycin-resistan* OR clarithromycin-nonsusceptible OR clarithromycin-non-susceptib*)):ti,ab)<br>OR<br>(((Campylobacter) AND (fluoroquinolone-resistan* OR fluorinated-quinolone-resistan* OR fluoro-quinolone-resistan* OR fluoroquinolones-resistan* OR fluoroquinolone-nonsusceptible OR fluoroquinolone-non-susceptible)):ti,ab)<br>OR<br>(((Salmonella) AND (fluoroquinolone-resistan* OR fluorinated-quinolone-resistan* OR fluoro-quinolone-resistan* OR fluoroquinolones-resistan* OR fluoroquinolone-nonsusceptible OR fluoroquinolone-non-susceptible)):ti,ab)<br>OR<br>(((Neisseria-gonorrhoeae OR Gonococcus-neisseri OR Gonococcus-neisseria OR Micrococcus-gonorrhoeae OR N.-gonorrhoeae OR Neisseria-gonococcus OR Neisseria-gonorrhaeae OR Neisseria-gonorrhea OR Neisseria-gonorrheae OR Neisseria-gonorrhoea) AND (3rd-generation-cephalosporin-resistan* OR third-generation-cephalosporin-resistan* OR fluoroquinolone-resistan* OR fluorinated-quinolone-resistan* OR fluoro-quinolone-resistan* OR fluoroquinolones-resistan* OR fluoroquinolone-nonsusceptible OR fluoroquinolone-non-susceptible)):ti,ab)<br>OR<br>('penicillin resistant Streptococcus pneumoniae'/exp OR ((Streptococcus-pneumoniae OR Diplococcus-pneumoniae OR Micrococcus-pneumoniae OR Pneumococcus OR pneumococcus OR Pneumococcus-pneumoniae) AND (penicillin-resistan* OR penicillin-non-susceptib* OR penicillin-nonsusceptib* OR fluoroquinolone-nonsusceptible OR fluoroquinolone-non-susceptible)):ti,ab)<br>OR	

For peer review only

	(((Haemophilus-influenza* OR Hemophilus-influenza* OR H.-influenza* OR Bacterium-influenza* OR Coccobacillus-pfeifferi OR Hemophilus-influenzae OR influenza-bacillus OR Mycobacterium-influenzae OR pfeiffer-bacillus) AND (ampicillin-resistan* OR Amp-resistan* OR ampicillin-nonsusceptib* OR ampicillin-non-susceptib*)):ti,ab)<br>OR<br>(((Shigella) AND (fluoroquinolone-resistan* OR fluorinated-quinolone-resistan* OR fluoro-quinolone-resistan* OR fluoroquinolones-resistan* OR fluoroquinolone-nonsusceptible OR fluoroquinolone-non-susceptible)):ti,ab)<br>OR<br>('multidrug resistant tuberculosis'/exp OR (RR-TB OR MDR-TB OR Pre-XDR-TB OR XDR-TB OR drug-resistant-TB OR multidrug-resistant-TB OR multidrug-resistant-tuberculos* OR drug-resistant-tuberculos* OR ((Mycobacterium-tuberculos* OR pulmonary-tuberculos* OR pulmonary-TB) AND (isoniazid-resistan* OR rifampicin-resistan* OR floroquinolone-resistan* OR levofloxacin-resistan* OR moxifloxacin-resistan* OR bedaquiline-resistan* OR linezolid-resistan* OR multidrug-resistan* OR drug-resistan*))):ab,ti)<br>OR<br>(((Aspergillus OR a.-fumigatus OR a.-flavus OR Neosartorya-fumigate OR Candida OR c.-auris OR c.-albicans) AND (resistan* OR non-susceptib* OR nonsusceptib*)):ti,ab)	
**#2 -** SR	('systematic review'/de OR 'meta-analysis'/de OR ((systematic* NEAR/3 review*) OR metaanaly* OR meta-analy* OR scoping):ab,ti)	564,680
**#3**	#1 AND #2	7,288
**#4 –** Animal Studies filter	NOT ('animal'/exp OR 'nonhuman'/exp NOT ('animal'/exp OR 'nonhuman'/exp AND 'human'/exp))	6,949
**#5** Specific animals	NOT (rat OR rats OR mouse OR mice OR hamster OR hamsters OR dog OR dogs OR cat OR cats OR bovine OR sheep):ti	6946
**#6-** Date filter	#5 AND [1-1-2012]/sd NOT [2-1-2022]/sd AND [2012-2022]/py	5,288

Pubmed
https://pubmed.ncbi.nlm.nih.gov/

#		**Results 10/12/2021**
**1** – AMR	("Drug Resistance, Bacterial"[Mesh] OR "Drug Resistance, Fungal"[Mesh] OR "Drug Resistance, Multiple, Bacterial"[Mesh] OR "Drug Resistance, Multiple, Fungal"[Mesh] OR "Antimicrobial Stewardship"[Mesh] OR drug-resistan*[tiab] OR multidrug-	318,242

For peer review only

	resistan*[tiab] OR anti-biotic-resistan*[tiab] OR antibiotic-resistan*[tiab] OR antibacterial-resistan*[tiab] OR anti-bacterial-resistan*[tiab] OR bacterial-resistan*[tiab] OR bacterium-resistan*[tiab] OR antimicrobial-resistan*[tiab] OR microbial-resistan*[tiab] OR anti-fungal-resistan*[tiab] OR antifungal-resistan*[tiab] OR cross-resistan*[tiab] OR antibiotic-non-susceptib*[tiab] OR antibiotic-nonsusceptib*[tiab] OR drug-non-susceptib*[tiab] OR drug-nonsusceptib*[tiab])<br>OR<br>((("Acinetobacter baumannii"[Mesh] OR Acinetobacter-baumannii*[tiab] OR Achromobacter-mucosus[tiab] OR Acinetobacter-baumanii[tiab] OR Acinetobacter-baumanni[tiab] OR Acinetobacter-genomosp.-2[tiab] OR Acinetobacter-genomospecies-2[tiab] OR Bacterium-anitratum[tiab] OR Bact.-anitratum[tiab]) AND (carbapenem-resistan*[tiab] OR carbapenem-non-susceptib*[tiab] OR carbapenem-nonsuscept*[tiab])))<br>OR<br>((("Pseudomonas aeruginosa"[Mesh] OR Pseudomonas-aeruginosa[tiab] OR Bacillus-aeruginosus[tiab] OR Bacillus-pyocyaneus[tiab] OR Bacterium-aeruginosum[tiab] OR Bacterium-pyocyaneum[tiab] OR blue-pus-organism[tiab] OR Micrococcus-pyocyaneus[tiab] OR P.-aeruginosa[tiab] OR Pseudomonas-polycolor[tiab] OR Pseudomonas-pyoceaneus[tiab] OR Pseudomonas-pyocyanea[tiab] OR Pseudomonas-pyocyaneus[tiab]) AND (carbapenem-resistan*[tiab] OR carbapenem-non-susceptib*[tiab] OR carbapenem-nonsuscept*[tiab])))<br>OR<br>(("Carbapenem-Resistant Enterobacteriaceae"[Mesh] OR ("Enterobacteriaceae"[Mesh] OR Enterobacteriaceae[tiab] OR enteric-bacteria[tiab] OR enterobacteria[tiab] OR enterobacteriacea[tiab] OR enterobacterium[tiab] OR Klebsiella-pneumonia[tiab] OR Escherichia-coli[tiab] OR Bacillus-coli[tiab] OR Bacillus-escherichii[tiab] OR Bacterium-coli[tiab] OR Bacterium-coli[tiab] OR bacterium-E3[tiab] OR coli-bacillus[tiab] OR coli-bacterium[tiab] OR colibacillus[tiab] OR colon-bacillus[tiab] OR E-coli[tiab] OR E.-coli[tiab] OR E.coli[tiab] OR Enterococcus-coli[tiab] OR Escherichia-alkalescens[tiab] OR Enterobacter[tiab] OR Serratia[tiab] OR Proteus[tiab] OR Providencia[tiab] OR Morganella[tiab]) AND (carbapenem-resistan*[tiab] OR carbapenem-non-susceptib*[tiab] OR carbapenem-nonsuscept*[tiab] OR 3rd-generation-cephalosporin-resistan*[tiab] OR third-generation-cephalosporin-resistan*[tiab] OR third-generation-cephalosporin-non-susceptib*[tiab])))<br>OR<br>("Vancomycin-Resistant Enterococci"[Mesh] OR VAN-R-enterococci OR VAN-R-Enterococcus OR<br>(("Enterococcus faecium"[Mesh] OR Streptococcus-faecium OR Enterococcus-faecium) AND ("Vancomycin Resistance"[Mesh] OR	

For peer review only

	vancomycin-resistan*[tiab] OR VAN-resistan*[tiab] OR vancomycin-non-susceptib*[tiab] OR vancomycin-nonsusceptib*[tiab]))) OR ("Methicillin-Resistant Staphylococcus aureus"[Mesh] OR "vancomycin resistant Staphylococcus aureus"[mesh] OR "Vancomycin-Resistant Staphylococcus aureus"[Mesh] OR MRSA[tiab] OR VRSA[tiab] OR (("Staphylococcus aureus"[Mesh] OR Staphylococcus-aureus[tiab] OR S.-aureus[tiab]) AND ("Methicillin Resistance"[Mesh] OR "Vancomycin Resistance"[Mesh] OR vancomycin-resistan*[tiab] OR VAN-resistan*[tiab] OR vancomycin-nonsusceptib*[tiab] OR vancomycin-non-susceptib*[tiab] OR Van-intermediate-resistan*[tiab] OR vancomycin-intermediate-resistan*[tiab] OR methicillin-resistan*[tiab] OR methicillin-nonsusceptib*[tiab]))) OR ((("Helicobacter pylori"[Mesh] OR Helicobacter-pylori[tiab] OR Campylobacter-pylori*[tiab] OR Helicobacter-nemestrinae) AND (clarithromycin-resistan* OR clarithromycin-non-susceptib*[tiab]))) OR ((("Campylobacter"[Mesh] OR Campylobacter[tiab]) AND (fluoroquinolone-resistan*[tiab] OR fluorinated-quinolone-resistan*[tiab] OR fluoro-quinolone-resistan*[tiab] OR fluoroquinolones-resistan*[tiab] OR fluoroquinolone-nonsusceptible[tiab] OR fluoroquinolone-non-susceptible[tiab]))) OR ((("Salmonella"[Mesh] OR Salmonella[tiab]) AND (fluoroquinolone-resistan*[tiab] OR fluorinated-quinolone-resistan*[tiab] OR fluoro-quinolone-resistan*[tiab] OR fluoroquinolones-resistan*[tiab] OR fluoroquinolone-nonsusceptible[tiab] OR fluoroquinolone-non-susceptible[tiab]))) OR ((("Neisseria gonorrhoeae"[Mesh] OR Neisseria-gonorrhoeae[tiab] OR Gonococcus-neisseri[tiab] OR Gonococcus-neisseria[tiab] OR Micrococcus-gonorrhoeae[tiab] OR N.-gonorrhoeae[tiab] OR Neisseria-gonococcus[tiab] OR Neisseria-gonorrhaeae[tiab] OR Neisseria-gonorrhea[tiab] OR Neisseria-gonorrheae[tiab] OR Neisseria-gonorrhoea[tiab]) AND (3rd-generation-cephalosporin-resistan*[tiab] OR third-generation-cephalosporin-resistan*[tiab] OR fluoroquinolone-resistan*[tiab] OR fluorinated-quinolone-resistan*[tiab] OR fluoro-quinolone-resistan*[tiab] OR fluoroquinolones-resistan*[tiab] OR fluoroquinolone-nonsusceptible[tiab] OR fluoroquinolone-non-susceptible[tiab]))) OR ((("Streptococcus pneumoniae"[Mesh] OR Streptococcus-pneumoniae[tiab] OR Diplococcus-pneumoniae[tiab] OR Micrococcus-pneumoniae[tiab] OR Pneumococcus[tiab] OR pneumococcus[tiab] OR Pneumococcus-pneumoniae[tiab]) AND	

For peer review only

	("Penicillin Resistance"[Mesh] OR penicillin-resistan*[tiab] OR penicillin-non-susceptib*[tiab] OR penicillin-nonsusceptib*[tiab]))) OR ((("Haemophilus influenzae"[Mesh] OR Hemophilus-influenza*[tiab] OR Bacterium-influenzae OR Coccobacillus-pfeifferi OR H.-influenza*[tiab] OR Haemophilus-influenza*[tiab] OR influenza-bacillus[tiab] OR Mycobacterium-influenzae[tiab] OR pfeiffer-bacillus[tiab]) AND (ampicillin-resistan*[tiab] OR Amp-resistan*[tiab] OR ampicillin-nonsusceptib*[tiab] OR ampicillin-non-susceptib*[tiab]))) OR ((("Shigella"[Mesh] OR Shigella[tiab]) AND (fluoroquinolone-resistan*[tiab] OR fluorinated-quinolone-resistan*[tiab] OR fluoro-quinolone-resistan*[tiab] OR fluoroquinolones-resistan*[tiab] OR fluoroquinolone-nonsusceptible[tiab] OR fluoroquinolone-non-susceptible[tiab]))) OR ("Tuberculosis, Multidrug-Resistant"[Mesh] OR "Extensively Drug-Resistant Tuberculosis"[Mesh] OR RR-TB OR MDR-TB OR Pre-XDR-TB OR XDR-TB OR drug-resistant-TB OR multidrug-resistant-TB OR multidrug-resistant-tuberculos*[tiab] OR drug-resistant-tuberculos*[tiab] OR ((Mycobacterium-tuberculos*[tiab] OR pulmonary-tuberculos*[tiab] OR pulmonary-TB[tiab]) AND (isoniazid-resistan*[tiab] OR rifampicin-resistan*[tiab] OR floroquinolone-resistan*[tiab] OR levofloxacin-resistan*[tiab] OR moxifloxacin-resistan*[tiab] OR bedaquiline-resistan*[tiab] OR linezolid-resistan*[tiab] OR multidrug-resistan*[tiab] OR drug-resistan*[tiab]))) OR ((("Aspergillus"[Mesh] OR Aspergillus[tiab] OR a.-fumigatus[tiab] OR a.-flavus[tiab] OR Neosartorya-fumigate[tiab] OR Candida[tiab] OR c.-auris[tiab] OR c.-albicans[tiab]) AND (resistan*[tiab] OR non-susceptib*[tiab] OR nonsusceptib*[tiab])))	
**#2 -** SR	"Systematic Reviews as Topic"[Mesh] OR "Systematic Review" [Pt] OR systematic[sb] OR systematic-review*[tiab] OR meta-analysis[pt] OR meta-analysis as topic[mh] OR metaanalys*[tiab] OR meta-analys*[tiab] OR scoping[tiab]	366,960
**#3**	#1 AND #2	3,887
**#4 –** Animal Studies filter	NOT ("Animals"[Mesh] NOT ("Animals"[Mesh] AND "Humans"[Mesh]))	3,845
**#5** Specific animals	NOT (rat[ti] OR rats[ti] OR mouse[ti] OR mice[ti] OR hamster[ti] OR hamsters[ti] OR dog[ti] OR dogs[ti] OR cat[ti] OR cats[ti] OR bovine[ti] OR sheep[ti])	3,839
**#6-** Date filter	#5 AND [1-1-2012]/sd NOT [2-1-2022]/sd AND [2012-2022]/py	3,181

For peer review only

Web of Science
https://www.webofscience.com/

#		Results 06/12/2021
**1** – AMR	(drug-resistan* OR multidrug-resistan* OR anti-biotic-resistan* OR antibiotic-resistan* OR antibacterial-resistan* OR anti-bacterial-resistan* OR bacterial-resistan* OR bacterium-resistan* OR antimicrobial-resistan* OR microbial-resistan* OR anti-fungal-resistan* OR antifungal-resistan* OR cross-resistan* OR antibiotic-non-susceptib* OR antibiotic-nonsusceptib* OR drug-non-susceptib* OR drug-nonsusceptib*)<br>OR<br>(((Acinetobacter-baumannii* OR Achromobacter-mucosus OR Acinetobacter-baumanii OR Acinetobacter-baumanni OR Acinetobacter-genomosp.-2 OR Acinetobacter-genomospecies-2 OR Bacterium-anitratum OR Bact.-anitratum) AND (carbapenem-resistan* OR carbapenem-non-susceptib* OR carbapenem-nonsuscept*)))<br>OR<br>(((Pseudomonas-aeruginosa OR Bacillus-aeruginosus OR Bacillus-pyocyaneus OR Bacterium-aeruginosum OR Bacterium-pyocyaneum OR blue-pus-organism OR Micrococcus-pyocyaneus OR P.-aeruginosa OR Pseudomonas-polycolor OR Pseudomonas-pyoceaneus OR Pseudomonas-pyocyanea OR Pseudomonas-pyocyaneus) AND (carbapenem-resistan* OR carbapenem-non-susceptib* OR carbapenem-nonsuscept*)))<br>OR<br>(((Enterobacteriaceae OR enteric-bacteria OR enterobacteria OR enterobacteriacea OR enterobacterium OR Klebsiella-pneumonia OR Escherichia-coli OR Bacillus-coli OR Bacillus-escherichii OR Bacterium-coli OR Bacterium-coli OR bacterium-E3 OR coli-bacillus OR coli-bacterium OR colibacillus OR colon-bacillus OR E-coli OR E.-coli OR E.coli OR Enterococcus-coli OR Escherichia-alkalescens OR Enterobacter OR Serratia OR Proteus OR Providencia OR Morganella) AND (carbapenem-resistan* OR carbapenem-non-susceptib* OR carbapenem-nonsuscept* OR 3rd-generation-cephalosporin-resistan* OR third-generation-cephalosporin-resistan* OR third-generation-cephalosporin-non-susceptib*)))<br>OR<br>(VAN-R-enterococci OR VAN-R-Enterococcus OR ((Streptococcus-faecium OR Enterococcus-faecium) AND (vancomycin-resistan* OR VAN-resistan* OR vancomycin-non-susceptib* OR vancomycin-nonsusceptib*)))<br>OR<br>(MRSA OR VRSA OR ((Staphylococcus-aureus OR S.-aureus) AND (vancomycin-resistan* OR VAN-resistan* OR vancomycin-	339,733

For peer review only

	nonsusceptib* OR vancomycin-non-susceptib* OR Van-intermediate OR vancomycin-intermediate OR methicillin-resistan*)))<br>OR<br>(((Helicobacter-pylori OR Campylobacter-pylori* OR Helicobacter-nemestrinae) AND (clarithromycin-resistan* OR clarithromycin-non-susceptib*)))<br>OR<br>(((Campylobacter) AND (fluoroquinolone-resistan* OR fluorinated-quinolone-resistan* OR fluoro-quinolone-resistan* OR fluoroquinolones-resistan* OR fluoroquinolone-nonsusceptible OR fluoroquinolone-non-susceptible)))<br>OR<br>(((Salmonella) AND (fluoroquinolone-resistan* OR fluorinated-quinolone-resistan* OR fluoro-quinolone-resistan* OR fluoroquinolones-resistan* OR fluoroquinolone-nonsusceptible OR fluoroquinolone-non-susceptible)))<br>OR<br>(((Neisseria-gonorrhoeae OR Gonococcus-neisseri OR Gonococcus-neisseria OR Micrococcus-gonorrhoeae OR N.-gonorrhoeae OR Neisseria-gonococcus OR Neisseria-gonorrhaeae OR Neisseria-gonorrhea OR Neisseria-gonorrheae OR Neisseria-gonorrhoea) AND (3rd-generation-cephalosporin-resistan* OR third-generation-cephalosporin-resistan* OR fluoroquinolone-resistan* OR fluorinated-quinolone-resistan* OR fluoro-quinolone-resistan* OR fluoroquinolones-resistan* OR fluoroquinolone-nonsusceptible OR fluoroquinolone-non-susceptible)))<br>OR<br>(((Streptococcus-pneumoniae OR Diplococcus-pneumoniae OR Micrococcus-pneumoniae OR Pneumococcus OR pneumococcus OR Pneumococcus-pneumoniae) AND (penicillin-resistan* OR penicillin-non-susceptib* OR penicillin-nonsusceptib*)))<br>OR<br>(((Hemophilus-influenza* OR Bacterium-influenzae OR Coccobacillus-pfeifferi OR H.-influenza* OR Haemophilus-influenza* OR influenza-bacillus OR Mycobacterium-influenzae OR pfeiffer-bacillus) AND (ampicillin-resistan* OR Amp-resistan* OR ampicillin-nonsusceptib* OR ampicillin-non-susceptib*)))<br>OR<br>(((Shigella) AND (fluoroquinolone-resistan* OR fluorinated-quinolone-resistan* OR fluoro-quinolone-resistan* OR fluoroquinolones-resistan* OR fluoroquinolone-nonsusceptible OR fluoroquinolone-non-susceptible)))<br>OR<br>(RR-TB OR MDR-TB OR Pre-XDR-TB OR XDR-TB OR drug-resistant-TB OR multidrug-resistant-TB OR multidrug-resistant-tuberculos* OR drug-resistant-tuberculos* OR ((Mycobacterium-tuberculos* OR pulmonary-tuberculos* OR pulmonary-TB) AND	

For peer review only

	(isoniazid-resistan* OR rifampicin-resistan* OR floroquinolone-resistan* OR levofloxacin-resistan* OR moxifloxacin-resistan* OR bedaquiline-resistan* OR linezolid-resistan* OR multidrug-resistan* OR drug-resistan*)))<br>OR<br>(((Aspergillus OR a.-fumigatus OR a.-flavus OR Neosartorya-fumigate OR Candida OR c.-auris OR c.-albicans) AND (resistan* OR non-susceptib*)))	
**#2 -** SR	TS=("Systematic Review" OR (systematic NEAR/3 *review*) OR metaanalys* OR meta-analys* OR met*analys* OR (scoping NEAR/3 (literature OR *review*)))	602,250
**#3**	#1 AND #2	5,279
**#4** Specific animals	NOT TI=(rat OR rats OR mouse OR mice OR hamster OR hamsters OR dog OR dogs OR cat OR cats OR bovine OR sheep)	5,254
**#5-** Date filter	#5 AND Timespan: 2012-01-01 to 2022-01-01 (Index Date)	4,428

For peer review only

Supplementary Table 2. Data attributes to be extracted

**Document-level attributes**	
Author(s);	
Author type	- Government agency;<br>- International (technical) agency/organization;<br>- Funding agency/donor;<br>- Research organization/university;<br>- Expert group/professional association;<br>- Individual authors;<br>- Audit/accreditation body;<br>- Private for-profit organization;<br>- Non-government organization;<br>- Other<br>- Unassigned
Journal/Book	Open text
Publisher	Open text
Publication year	
Country/countries of origin	Open text
Funding source	Open text
Information source	- WHO document;<br>- WHO guideline;<br>- Grey literature;<br>- Systematic reviews in the bibliographic database;<br>- Scientific papers suggested by the WHO AMR Steering Group
Purpose/Type of document	- Guidance/guideline;<br>- Report;<br>- Systematic review/meta-analysis;<br>- Narrative review/commentary/editorial;<br>- Other
Data extractor 1 and 2	
Date of data extraction 1 and 2	
Confirmation on document's inclusion/exclusion criteria	Inclusion criteria:<br>1. Document describes one or more knowledge gaps or research questions relevant to AMR, including priorities, framework, components, elements, or steps for the description (i.e., epidemiology, burden and drivers), delivery, development and/or discovery of tools, products, or interventions for AMR prevention, diagnosis and/or care & treatment.<br>2. Document has a global or regional application and/or relevance for LMIC.<br>Exclusion criteria:

For peer review only

	1. Document only describes individual research studies or case reports;<br>2. Related to microorganisms not included in the WHO priority pathogen list, TB or critical fungi (e.g., parasites, viruses);<br>3. Non-human sectors;<br>4. Document has no identifiable authors, publisher and year of publication.
**Document-level and research-question level attributes**	
Geographical scope	- Unassigned<br>- Global<br>- Regional;<br>- National;<br>- Subnational/local
Region (if applicable)	Open text;
Country/-ies (if applicable)	Open text;
Resource/socioeconomic context (World Bank definition)	- Unassigned<br>- General/overall<br>- LIC<br>- MIC<br>- HIC<br>- Low- and Middle-Income Countries (LMIC)
Population	- Unassigned<br>- General/overall<br>- Adult<br>- Children<br>- Neonates<br>- Elderly<br>- Immunocompromised<br>- Comorbidities<br>- Pregnant women<br>- Other vulnerable groups
Setting	- Unassigned<br>- General/overall<br>- Community<br>- Health care system<br>o Primary health care<br>o Acute-care hospitals (including secondary and tertiary care)<br>o Long-term care facilities (including nursing homes)<br>o ICU
Micro-organism group	- Unassigned<br>- General/overall<br>- PPL<br>- TB<br>- Critical fungi

For peer review only

Antibiotic-microorganism combination (refer to Table 1)<br>-	- Unassigned<br>- General/overall<br>- Acinetobacter baumannii: carbapenem-resistant<br>- Pseudomonas aeruginosa: carbapenem-resistant<br>- Enterobacteriaceae (including Klebsiella pneumonia, Escherichia coli, Enterobacter spp., Serratia spp., Proteus spp., and Providencia spp, Morganella spp.): carbapenem-resistant, 3rd generation cephalosporin-resistant<br>- Enterococcus faecium: vancomycin-resistant<br>- Staphylococcus aureus: methicillin-resistant, vancomycin-intermediate and -resistant<br>- Helicobacter pylori: clarithromycin-resistant<br>- Campylobacter: fluoroquinolone-resistant<br>- Salmonella spp.: fluoroquinolone-resistant<br>- Neisseria gonorrhoeae: 3rd generation cephalosporin-resistant, fluoroquinolone-resistant<br>- Streptococcus pneumoniae: penicillin-non-susceptible<br>- Haemophilus influenzae: ampicillin-resistant<br>- Shigella spp.: fluoroquinolone-resistant<br>- RR-TB<br>- MDR-TB<br>- Pre-XDR-TB<br>- XDR-TB<br>- Aspergillus spp (including fumigatus and flavus)<br>- Candida spp. (including albicans and auris).
Syndrome	- Unassigned<br>- General/overall<br>- Bloodstream infection/sepsis<br>- Pneumonia/respiratory tract<br>- Gastrointestinal infection<br>- Urinary tract infection<br>- Skin and soft tissue<br>- Central nervous system infection<br>- Sexually Transmitted Infection<br>- TB-general/unspecified<br>- TB-pulmonary<br>- TB-extrapulmonary<br>- Other
Sector	- Human health sector<br>- One health (intersection/overlapping between animal, environment and human health sector)
Topic	- Unassigned<br>- General/overall<br>- AMR National Action Plans and Monitoring & Evaluation<br>- AMR surveillance<br>- Antimicrobial consumption surveillance<br>- AMR One Health

For peer review only

	- Antimicrobial Use/AMR Stewardship/Essential Medicines<br>- Antimicrobial R&D<br>- AMR awareness<br>- Diagnostics (including R&D)<br>- Infection Prevention and Control<br>- Water, Sanitation, Hygiene and Health<br>- Immunization/Vaccine (including Product & Delivery Research)<br>- Food safety/food borne diseases<br>- Sexually Transmitted Infections<br>- TB Prevention, Diagnosis, Treatment, Care & Innovation<br>- Newborn health<br>- Health systems strengthening/Universal Health Coverage/Policy and regulatory
**Research-question level attributes**	
Theme	- Prevention (subcategories for CAI and HAI, immunization, IPC, other)<br>- Diagnosis<br>- Care & treatment (subcategories: CAI and HAI)<br>- Unassigned
Domain	- Descriptive (subcategories: measuring the burden; understanding risk factors; measuring prevalence of exposure to risk factors; Evaluating the efficacy and effectiveness of interventions in place; measuring prevalence of coverage of interventions in place)<br>- Delivery (subcategories: Health policy analysis; Health system structure analysis; Financing/costs analysis; Human resources; Provision/infrastructure; Operations research; Responsiveness/recipients)<br>- Development (subcategories: acceptability, affordability, deliverability/feasibility, sustainability)<br>- Discovery (subcategories: medicines, diagnostics, vaccines, digital health technology, other preventive tools, other)<br>- Unassigned
Area	- Epidemiology (including drivers, prevalence and burden)<br>- Pharmacological and clinical<br>- Behavioural and social science<br>- Health economics/cost-effectiveness<br>- Legislation and regulation

Abbreviations: AMR: antimicrobial resistance; CAI: community-acquired infection; HAI: healthcare-associated infection; HIC: high-income country; ICU: intensive care unit; IPC: infection prevention and control; LIC: low-income country; LMIC: low- and middle-income country; MIC: middle-income country MDR-TB: multidrug-resistant tuberculosis; PPL: WHO priority pathogen list; Pre-XDR-TB: pre-extensively drug-resistant tuberculosis; RR-TB: rifampicin-resistant tuberculosis; R&D: research and development; TB: tuberculosis; XDR-TB: extensively drug-resistant tuberculosis

For peer review only

For peer review only

BMJ Open

Developing a priority global research agenda for antimicrobial resistance in the human health sector: protocol for a scoping review

Journal:	*BMJ Open*
Manuscript ID	bmjopen-2021-060553.R1
Article Type:	Protocol
Date Submitted by the Author:	29-Mar-2022
Complete List of Authors:	Hamers, Raph Leonardus; University of Oxford Medical Sciences Division, Eijkman-Oxford Clinical Research Unit; University of Oxford Medical Sciences Division, Nuffield Department of Medicine<br>Cassini, Alessandro; World Health Organization, Department of Control, Surveillance and Prevention of Antimicrobial Resistance<br>Asadinia, Koe Stella; University of Oxford, Eijkman-Oxford Clinical Research Unit<br>Bertagnolio, S; World Health Organization, Department of Control, Surveillance and Prevention of Antimicrobial Resistance
**Primary Subject Heading**:	Global health
Secondary Subject Heading:	Global health, Infectious diseases
Keywords:	INFECTIOUS DISEASES, Diagnostic microbiology < INFECTIOUS DISEASES, Epidemiology < INFECTIOUS DISEASES, Infection control < INFECTIOUS DISEASES, Public health < INFECTIOUS DISEASES

SCHOLARONE™
Manuscripts

Developing a priority global research agenda for antimicrobial resistance in the human health sector: protocol for a scoping review

Authors

Raph L. Hamers[1,2], Alessandro Cassini[3], Koe Stella Asadinia[1], Silvia Bertagnolio[3]

Affiliations

1. Eijkman-Oxford Clinical Research Unit, Jakarta, Indonesia;
2. Centre for Tropical Medicine and Global Health, Nuffield Department of Medicine, University of Oxford, Oxford, UK;
3. Control and Response Strategies (CRS) Unit, Department of Control, Surveillance and Prevention of Antimicrobial Resistance, World Health Organization, Geneva, Switzerland

Correspondence to

Silvia Bertagnolio, MD

Control and Response Strategies (CRS) Unit

Department of Control, Surveillance and Prevention of Antimicrobial Resistance

World Health Organization

20, Avenue Appia

1211 Geneva 27, Switzerland

Mobile: +41 79 7288078

Office +41 22 791 3958

Email: bertagnolios@who.int

Word count: 2505

Inserts Tables 3**;** Panel 1

Supplemental material: Tables 2

For peer review only

Abstract

Introduction To accelerate the response to the public health threat by antimicrobial resistance (AMR), the World Health Organization (WHO) is developing a Global Research Agenda for AMR in the human health sector that aims to provide a global and transparent assessment of priority knowledge gaps related to critical bacteria – including *Mycobacterium tuberculosis*– and fungi that inform control and response strategies to tackle AMR by 2030. A literature scoping review represents the first phase in a stepwise process, and we hereby outline the protocol to review current knowledge gaps and research questions on AMR in the human health sector.

Methods and analysis This literature scoping review will follow the Arksey and O'Malley (2005) methodology and will include: i) a hand search to identify relevant WHO guidelines and documents suggested by the WHO Steering Group for the AMR Global Research Agenda; ii) a grey literature search through a stakeholder mapping process and google searches of organisational websites; iii) a systematic search of relevant systematic reviews through bibliographic databases (PubMed, Embase and Web of Science); iv) screening of the reference lists of included studies. We will include relevant publications from the last 10 years (January 2012-December 2021). Two researchers separately will review the yielded citations to determine eligibility based on pre-defined criteria. Relevant research questions with attributes will be extracted using a tool developed through an iterative process by the research team. Each identified research question will be classified and aggregated according to a conceptual framework (i.e. "knowledge matrix"), composed of three themes (i.e. Prevention, Diagnosis and Care & Treatment) and four cross-cutting domains (i.e. Descriptive, Discovery, Development, Delivery). We will present numerical and thematic summaries of the knowledge matrix. A qualitative content analysis is out of the scope of this protocol.

Ethics and dissemination The scoping review process will only involve identification, selection and analysis of documents available for use in the public

For peer review only - http://bmjopen.bmj.com/site/about/guidelines.xhtml

domain, and will not include any personal information on individuals, therefore ethical approval is not required. The findings will be disseminated through a peer-reviewed publication and stakeholder meetings.

Keywords antimicrobial resistance; knowledge gaps, research gaps, research questions

Article Summary: Strengths and limitations of this study

- This is a novel literature scoping review that aims to identify knowledge gaps and research questions on AMR, as the first phase in a stepwise process for developing a priority AMR Global Research Agenda, in consultation with the WHO Steering Group for the AMR Global Research Agenda, and a large group of external experts, policy makers and stakeholders.
- Adopting the modified standardised method for setting priorities in health research developed by the Child Health and Nutrition Research Initiative (CHNRI), the scoping review will populate a pre-defined conceptual framework ("knowledge matrix") to capture and categorize research questions related to the description of AMR burden and drivers, and the delivery, development and/or discovery of interventions for AMR prevention, diagnosis and/or care & treatment.
- The identification and synthesis of data will include WHO documents, grey literature and systematic reviews published in 2012-2021, to capture the most important research questions and knowledge gaps and inform the further consultation process.

For peer review only - http://bmjopen.bmj.com/site/about/guidelines.xhtml

INTRODUCTION

Background

The global rise in antimicrobial resistance (AMR) is considered one of the greatest public health threats, with a growing and disproportionate impact in low- and middle-income countries (LMIC).[1] One of the major drivers of AMR is antimicrobial misuse and overuse both in humans and animals for human consumption.[2] Lack of access to clean water and sanitation in low-resource settings and inadequate infection prevention and control promotes the spread of microbes, including those resistant to antimicrobial treatment. Access to life-saving drugs targeting the emerging resistant pathogens remains an issue in LMIC, while much has been written about the dire state of the pipeline of new antibacterial drugs. These realities occur in a wider context of lack of access to quality health care in many settings. Key strategies to address AMR are to reduce the need for antibiotics (through prevention of infections), to reduce the use of antibiotics (by better use of diagnostics and antibiotic stewardship), and to find new approaches for disease prevention and treatment.

To address the serious public health threat posed by AMR, WHO coordinated the development of a Global Action Plan (GAP) on AMR in 2015, which in turn has been translated into National Action Plans.[3] To accelerate their implementation, there is a need to expand the evidence-base on AMR burden, impact and interventions, including their prioritization, cost-effectiveness, financing and knowledge on how to bring existing interventions at scale.[4–6] Since their implementation has been further set back due to the COVID-19 pandemic, now more than ever, there is an urgent need to revitalize work on AMR to control this silent pandemic.[7] Appropriate targeted research is needed to make substantial progress in attaining the 2030

For peer review only

Sustainable Development Goals (SDGs) and to provide the evidence-base in support of successful implementation of interventions to prevent, diagnose and manage drug-resistant infections.

The WHO is developing a Global Research Agenda for AMR that aims to provide a global and transparent assessment of knowledge gaps related to prevention and control of resistant bacterial –including M. tuberculosis– and fungal infections in the human health sector. This agenda is expected to catalyse investment and focus scientific interest among researchers, donors and public health professionals towards the generation of new and critical evidence to address knowledge gaps for effective implementation of global, regional and national policies on AMR, particularly LMIC, in the timeframe 2023-2030. The evidence generated will inform prioritization of interventions within AMR National Action Plans. This work focuses on the human health sector only, and will complement the WHO One Health Priority Research Agenda for AMR, being developed in parallel, which focuses on the interface between humans, animals, plants and their shared environment. The first step in the stepwise development of the Agenda is to conduct a literature scoping literature review to identify existing research questions and knowledge gaps on AMR; this paper outlines the protocol of the scoping review.

Aim and Objectives

To conduct a scoping literature review to identify existing research questions and knowledge gaps on AMR in the human health sector focusing on:

- Burden of resistant bacterial (including *M. tuberculosis*) and fungal infections and factors associated with and predictive of AMR emergence and transmission;

For peer review only

For peer review only - http://bmjopen.bmj.com/site/about/guidelines.xhtml

- New or improved interventions, technologies and tools that are associated with improved AMR prevention, diagnosis, care and treatment and best ways to deliver these

METHODS AND ANALYSIS

Conceptual framework and scope

The development of the AMR Global Research Agenda will follow the WHO methodological framework *Guidance for undertaking a research priority setting exercise*,[8] which includes a modified version of the standardised method for setting priorities in health research developed by the Child Health and Nutrition Research Initiative (CHNRI).[9,10] The CHNRI framework proposes a flexible, systematic approach to listing a large number of research options and research questions, and allows for a prioritisation exercise that is systematic, transparent, legitimate and fair, scientifically rigorous and replicable. Evidence needed should be clearly framed by the research options or questions, and the effect of its translation and implementation into interventions should be straightforward. The CHNRI framework uses the “4D framework” of “description”, “delivery”, “development” and “discovery” research.[9,10] Each identified research question will thus be classified and aggregated to populate a “knowledge matrix”, composed of three focus areas or "themes" (i.e., Prevention, Diagnosis and Care & Treatment) and four cross-cutting domains (Descriptive, Discovery, Development, Delivery), as described in **Table 1**.

Table 1. Knowledge matrix

		Themes		
		**Prevention**	**Diagnosis**	**Care & Treatment**
**Cross-cutting**	**Descriptive**			
	**Delivery**			

For peer review only

**domains**	**Development**			
**(“4Ds”)**	**Discovery**			

Table footnote: Definitions	
Theme/Domain	Description
Prevention	Prevention of AMR in hospital- and community-acquired infections
Diagnosis	Diagnosis of infections caused by microorganisms resistant to antimicrobials, including both pathogen identification and antibiotic susceptibility (AMR) testing
Care & treatment	Care and treatment of hospital- and community-acquired infections caused by antimicrobial-resistant micro-organisms
Descriptive	Improve understanding of AMR epidemiology, burden and factors associated with and predictive of AMR emergence and transmission (epidemiological research)
Delivery of existing interventions	Optimize and improve the delivery of existing interventions (implementation research, operations research, and/or health policy and systems research)
Development of improved interventions	Improve existing interventions by reducing costs, or optimizing uptake, impact, sustainability and feasibility
Discovery of new interventions	New tools and interventions (new medicines, diagnostics, vaccines, or other interventions)

Abbreviations: AMR: antimicrobial resistance

For peer review only

To populate the “knowledge matrix” as a first step in this iterative process, we will conduct a scoping review of existing research questions through reviews of published and grey literature. The scoping review will also be informed by inputs from the WHO Steering Group for the AMR Global Research Agenda (SG) composed of technical experts in areas relevant to AMR, including AMR National Action Plans, Monitoring & Evaluation; surveillance and antimicrobial use; stewardship and awareness; antimicrobial research and development (R&D); WHO essential medicines; diagnostics; infection prevention and control (IPC); water, sanitation, hygiene and health (WASH); vaccine/product & delivery research; the Special Programme for Research and Training In Tropical Diseases (TDR); food safety/foodborne diseases; sexually transmitted infections; newborn health; and tuberculosis (TB) prevention, diagnosis, treatment, care & innovation. The scope of the scoping review is summarized in **Panel**.

Panel: Scope of the scoping review

The scoping review:

- Is global in its scope;

- Focuses exclusively on AMR in the human health sector;

- Focuses on priority research questions that can and should be addressed in the timeframe 2023-2030 (SDGs);

- Includes relevant publications published in the past 10 years (covering the period leading up to the launch of the Global Action Plan for AMR in 2015, and thereafter) [3];

- Is based on a “knowledge matrix” composed of three focus areas or "themes" (Prevention, Diagnosis and Care & Treatment) and four cross-cutting domains (Descriptive, Discovery, Development, Delivery)

- The following disaggregation is considered when categorizing the research questions:

  - Populations (e.g., adults, children, neonates, vulnerable groups);

For peer review only - http://bmjopen.bmj.com/site/about/guidelines.xhtml

- Settings (e.g., community, primary care, hospitals);
- Geographical/socioeconomic context (e.g., low- and middle-income countries; high-income countries)
- Syndrome (e.g., bloodstream infections, etc)
- Antibiotic-microorganism (“drug-bug”) combinations

- Will not rank or prioritize any of the included pathogens for the purpose of this review (see below);

- Focuses on the WHO Global priority list of antibiotic-resistant bacteria [11]:

- *Acinetobacter baumannii*: carbapenem-resistant
- *Pseudomonas aeruginosa*: carbapenem-resistant
- *Enterobacteriaceae* (including *Klebsiella pneumonia, Escherichia coli, Enterobacter spp., Serratia spp., Proteus spp.,* and *Providencia spp, Morganella spp.*): carbapenem-resistant, 3rd generation cephalosporin-resistant
- *Enterococcus faecium*: vancomycin-resistant
- *Staphylococcus aureus*: methicillin-resistant, vancomycin-intermediate and -resistant
- *Helicobacter pylori*: clarithromycin-resistant
- *Campylobacter*: fluoroquinolone-resistant
- *Salmonella spp.*: fluoroquinolone-resistant
- *Neisseria gonorrhoeae*: 3rd generation cephalosporin-resistant, fluoroquinolone-resistant
- *Streptococcus pneumoniae*: penicillin-non-susceptible
- *Haemophilus influenzae*: ampicillin-resistant
- *Shigella spp.*: fluoroquinolone-resistant

- Focuses on drug resistant critical fungi, i.e.:

- *Aspergillus spp.* (especially *A. fumigatus* and *A. flavus*)
- *Candida spp.* (especially *C. albicans* and *C. auris*).

For peer review only

- Focuses on drug resistant *Mycobacterium tuberculosis*, with the following definitions:

- Rifampicin-resistant TB (RR-TB);
- Multi-drug-resistant TB (MDR-TB**):** resistance to at least isoniazid and rifampicin;
- Pre-extensively drug-resistant TB (pre-XDR-TB**):** MDR/RR-TB and also resistant to any fluoroquinolone.
- Extensively drug-resistant TB (XDR-TB): pre-XDR-TB and also resistant to at least one additional Group A drug (comprises levofloxacin, moxifloxacin, bedaquiline and linezolid).

Abbreviations: AMR, antimicrobial resistance; HIC: high-income country; LMIC, low- and middle-income countries; MDR-TB, multidrug-resistant tuberculosis; Pre-XDR-TB, pre-extensively drug-resistant tuberculosis; RR-TB, rifampicin-resistant tuberculosis; SDGs, sustainable development goals; XDR-TB, extensively drug-resistant tuberculosis.

Protocol design

This protocol follows the methodological framework for scoping reviews developed by Arksey and O'Malley [12] and Levac *et al*. [13] through six stages, as well as the guidance developed by the Joanna Briggs Institute to standardize the conduct of scoping reviews.[14] Due to the iterative nature of a scoping review, protocol deviations during the process may be necessary. Any discrepancies will be detailed and justified in the Methods section of the final report. The scoping review is expected to be conducted between November 2021 and April 2022, and includes the following 6 stages.

Stage 1: Defining the scope and main research question of the scoping review

The purpose of the scoping review is to map the literature and identify the key research questions and knowledge gaps that inform the research priority setting

For peer review only - http://bmjopen.bmj.com/site/about/guidelines.xhtml

exercise to develop the AMR Global Research Agenda. This scoping review addresses the following broad question: "What are the existing research questions and knowledge gaps on the description, delivery, development and/or discovery of interventions for AMR prevention, diagnosis and/or care & treatment in the human health sector, at a global level?"

Stage 2: Searching for relevant documents

We plan to search several information sources of published and grey literature to identify relevant documents as listed in **Table 2**.

Table 2. List of information sources to be searched for the scoping review

1. WHO-authored documents and research gaps identified through WHO guidelines processes in areas relevant to AMR
• Target: Relevant documents including strategies, global action plans, meeting reports, policy briefs, technical documents, resolutions.<br>• Approach: we will manually search the WHO Institutional Repository for Data Sharing (IRIS), Data Platform, The Global Health Observatory, and WHO Observatory for Health R&D.
2. Grey literature relevant to AMR
• Target: Relevant position papers, reports, strategies, guidelines, and evaluations from national or international agencies and AMR stakeholders.<br>• Approach: First, we will conduct a global mapping of stakeholders working with AMR policy, advocacy, innovation and research, surveillance, based on the ReAct reference,[15] organisational websites, and feedback from the WHO Steering Group for the AMR Global Research Agenda. Second, we will perform a targeted manual search of organisational websites. Third, we will supplement the search above with a

For peer review only

google search including the term "filetype:pdf" and "antimicrobial resistance" (and synonyms).
3. Published systematic reviews on AMR research gaps and priorities
• Target: relevant systematic reviews.<br>• Approach: systematic search of the relevant bibliographic databases, i.e., PubMed, Embase and Web of Science (Full search terms in **Supplementary Table 1**)
4. Documents suggested by WHO Steering Group for the AMR Global Research Agenda
• Target: relevant scientific papers, editorials, reviews and grey literature.<br>• Approach: targeted requests to the WHO Steering Group members to share all documents relevant to their specific field of work.

Abbreviations: AMR, antimicrobial resistance; R&D, research and development

All searches will be jointly developed and conducted by an experienced WHO librarian, the authors and content experts in the SG. We will manually search the grey literature in organisations' websites and google, and consider any relevant documents as suggested by the SG. The full search terms for the bibliographic databases are available in **Supplementary Table 1.** For the non-general data sources, we will use less restrictive terms to be able to identify relevant documents. We will search reference lists of included documents for any additional potentially relevant references. The searches will be restricted to the past 10 years (January 2012-December 2021) and the English language. After the searches, the SG will review the search results to determine if the documents identified represent all relevant resources.

For peer review only

Stage 3: Document selection

This stage will be an iterative process involving searching the literature, refining the search strategy, and reviewing articles for eligibility and inclusion. The study team will meet to discuss study inclusion and exclusion criteria at the beginning of the process, and will discuss challenges and uncertainties related to study selection along the review process. Unlike systematic reviews, in scoping reviews, inclusion and exclusion criteria may be adjusted during the search and selection process, once familiarity with the literature has been gained. For grey literature, a first reviewer will read all full-text articles for eligibility, and a second reviewer will separately read at least 20% of all full-text articles; if the agreement is <80%, the second reviewer will dual-read all full-text articles. For bibliographic databases, a first reviewer will screen all titles and abstracts for eligibility, and a second reviewer will separately screen at least 20% of all titles and abstracts; if the agreement is <80%, a second reviewer will dual-screen all titles and abstracts. Then, a first reviewer will read all included full-text articles for eligibility, and a second reviewer will separately read at least 20% of all full-text articles; if the agreement is <80%, a second reviewer will dual-read all full-text articles. We will resolve disagreements on study selection by consensus and discussion with other reviewers as needed. For grey literature, duplicate documents will be removed in Mendeley manually and using the automated tool. For bibliographic databases, duplicate documents will be removed in EndNote using the Bramer method.[16] Documents that are superseded with a more recent version are excluded (and the most recent is included).

Document inclusion criteria:

1. Document describes one or more knowledge gaps or research questions on AMR, including priorities, framework, components, elements, or steps for the description (i.e., epidemiology, burden and drivers), delivery, development and/or discovery of

For peer review only - http://bmjopen.bmj.com/site/about/guidelines.xhtml

tools, products, or interventions for AMR prevention, diagnosis and/or care & treatment.

2. Document has a global or regional application and/or relevance for LMIC.

Document exclusion criteria:

1. Document only describes individual research studies or case reports;
2. Document is not related to bacteria included in the WHO priority pathogen list, M. tuberculosis or critical fungi (e.g., parasites, viruses);
3. Document is related to AMR in the non-human sectors;
4. Document has no identifiable authors, publisher and year of publication.

Stage 4: Extracting the data

Data elements will be extracted from included documents using a data extraction form in Microsoft Excel, which will be jointly developed by the research team and updated during the iterative extraction process ("charting the data"). Two reviewers will independently test the data extraction form on 5-10 documents to ensure all relevant results are extracted and that their approach to extraction is consistent with the research question and purpose. The content of the included documents will be read in-depth to identify research questions and knowledge gaps within the scope of the scoping review. A single reviewer will extract the data, and translate each research gap, need or question into one or more research questions that are specific for the attributes expressed in the source text. We will use a "narrative review" or "descriptive analytical" method to extract information from each document. We will extract explicitly stated research questions, and we will also use relevant text extracts to formulate additional research questions. This includes results of previous research priorities exercises if available. Each research question will be annotated with relevant attributes (**Supplementary Table 2)**. The extracted research questions and annotations will be reviewed and refined by at least two other team members.

For peer review only - http://bmjopen.bmj.com/site/about/guidelines.xhtml

The research team will meet weekly to review the research questions identified and ensure data extraction is consistent with the research question and purpose. The whole exercise will result in a database of annotated research questions. Document authors will not be contacted.

Stage 5: Collating, summarizing and reporting results

To provide an overview of the breadth of the literature, we will present numerical and thematic summaries of the key characteristics and categories of included documents and research questions, using the thematic construction of the "knowledge framework" (**Table 2**). The results may be presented as a "map" of the data in a logical, diagrammatic, or tabular form, and/or in a descriptive format that aligns to the overall study purpose and scope of the review. We will seek to minimize redundancies by deleting and/or merging research questions into higher-level research avenues where appropriate and feasible. The plan for presenting the results will be further refined toward the end of the review when the researchers have gained the greatest awareness of the contents of the included studies. However, an in-depth qualitative content analysis or synthesis, and discussion of implications for future research, practice and policy are outside the scope of this scoping review. Furthermore, we will not perform a formal assessment of the methodological quality of the included documents as the purpose of this scoping review is to achieve a broad and in-depth description of existing research questions and knowledge gaps around AMR.

We will report in compliance with the PRISMA extension for Scoping Reviews (PRISMA-ScR) including a flow diagram for the scoping review process and a completed checklist.[17]

For peer review only

For peer review only - http://bmjopen.bmj.com/site/about/guidelines.xhtml

Stage 6: Consulting with stakeholders

The scoping review process will be conducted in regular consultation with the SG to inform and validate the results. Later in the agenda-setting process, the findings of the scoping review will form a basis for various rounds of expert and stakeholder consultations for ranking, prioritizing and validation towards further finalisation of the WHO Global Research Agenda for AMR.

Patient and public involvement statement

The patient and public were not involved in the development of this study protocol.

ETHICS AND DISSEMINATION

The scoping review process will only involve identification, selection and analysis of documents available for use in the public domain, and will not include any personal information on individuals, therefore ethical approval is not required. The scoping review will be done in consultation with the SG and disseminated through a peer-reviewed publication. This scoping review is the first step in a stepwise process for developing a priority AMR Global Research Agenda, which will be done in consultation with global, regional and national stakeholders who are the end-users of the generated knowledge and disseminated through a final report.

For peer review only

Authors' contributions

SB and AC conceptualised the project. RLH, AC and SB developed the protocol and search strategies. RLH drafted the manuscript, with critical input from AC, KSA and SB. KSA designed and tested the data extraction tools. All authors critically reviewed the paper for important intellectual content and approved the final version of the manuscript.

Acknowledgements

The authors would like to acknowledge the contributions and suggestions of the WHO Steering Group for the AMR Global Research Agenda in the Human Health Sector: Kitty Van Weezenbeek, Sarah Paulin, Barbara Tornimbene, Tine Jorgensen, Peter Beyer, Thomas Joseph, Benedetta Allegranzi, Nebiat Gebreselassie, Mateusz Hasso-Agopsowicz, Rob Terry, Benedikt Huttner, Bruce Gordon, Francis Moussy, Luz De Regil, Sachiyo Yoshida, Teodora Wi. The authors also acknowledge the contributions and suggestions of other WHO colleagues: Anand Balachandran, Verica Ivanovska, Kate Medlicott, Arno Muller, Maarten van der Heijden, Carmem Pessoa, and Matteo Zignol, and the support of the WHO librarians Tomas Allen and Kavita Kothari.

Funding statement

This work is part of the project entitled "Global Research Agenda for Antimicrobial Resistance in the Human Health Sector" initiated and funded by WHO (no award/grant number).

Competing interests statement

SB and AC are WHO employees. RLH is a consultant for this work and is also supported by the Wellcome Trust (106680/Z/14/Z). The views expressed are those

For peer review only

of the authors and not necessarily those of WHO, Wellcome Trust, or any of the institutions mentioned.

Data sharing statement: No additional data are available.

ORCID IDs:

Raph L. Hamers 0000-0002-5007-7896

Alessandro Cassini 0000-0002-7973-6445

Koe Stella Asadinia 0000-0003-4552-5014

Silvia Bertagnolio 0000-0001-8416-2581

For peer review only

References

1 Sriram A, Kalanxhi E, Kapoor G, *et al.* Center for Disease Dynamics, Economics & Policy, Washington DC. State of the world's antibiotics 2021: A global analysis of antimicrobial resistance and its drivers. 2021.

2 Klein EY, Van Boeckel TP, Martinez EM, *et al.* Global increase and geographic convergence in antibiotic consumption between 2000 and 2015. *Proc Natl Acad Sci* 2018;:201717295. doi:10.1073/pnas.1717295115

3 World Health Organisation. Global action plan on antimicrobial resistance. 2015. https://www.who.int/publications/i/item/9789241509763

4 Laxminarayan R, Van Boeckel T, Frost I, *et al.* The Lancet Infectious Diseases Commission on antimicrobial resistance: 6 years later. *Lancet Infect Dis* 2020;**20**:e51–60. doi:10.1016/S1473-3099(20)30003-7

5 Laxminarayan R, Duse A, Wattal C, *et al.* Antibiotic resistance-the need for global solutions. Lancet Infect. Dis. 2013;**13**:1057–98. doi:10.1016/S1473-3099(13)70318-9

6 Wernli D, Jørgensen PS, Parmley EJ, *et al.* Evidence for action: a One Health learning platform on interventions to tackle antimicrobial resistance. *Lancet Infect Dis* 2020;**20**:e307–11. doi:10.1016/S1473-3099(20)30392-3

7 Tomczyk S, Taylor A, Brown A, *et al.* Impact of the COVID-19 pandemic on the surveillance, prevention and control of antimicrobial resistance: a global survey. *J Antimicrob Chemother* Published Online First: 2 September 2021. doi:10.1093/jac/dkab300

8 Organization WH. *A systematic approach for undertaking a research priority-setting exercise: guidance for WHO staff*. Geneva PP - Geneva: : World Health Organization https://apps.who.int/iris/handle/10665/334408

9 Rudan I, Gibson JL, Ameratunga S, *et al.* Setting priorities in global child health research investments: guidelines for implementation of CHNRI method.

For peer review only

For peer review only - http://bmjopen.bmj.com/site/about/guidelines.xhtml

Croat Med J 2008;**49**:720–33. doi:10.3325/cmj.2008.49.720

10 Rudan I. Setting health research priorities using the CHNRI method: IV. Key conceptual advances. *J Glob Health* 2016;**6**:10501. doi:10.7189/jogh-06-010501

11 World Health Organization. Prioritization of pathogens to guide discovery, research and development of new antibiotics for drug-resistant bacterial infections, including tuberculosis. 2017.

12 Arksey H, O'Malley L. Scoping studies: towards a methodological framework. *Int J Soc Res Methodol* 2005;**8**:19–32. doi:10.1080/1364557032000119616

13 Levac D, Colquhoun H, O'Brien KK. Scoping studies: advancing the methodology. *Implement Sci* 2010;**5**:69. doi:10.1186/1748-5908-5-69

14 Peters MDJ, Godfrey CM, Khalil H, *et al.* Guidance for conducting systematic scoping reviews. *JBI Evid Implement* 2015;**13**.https://journals.lww.com/ijebh/Fulltext/2015/09000/Guidance_for_conducting_systematic_scoping_reviews.5.aspx

15 ReAct. AMR stakheolder mapping. A global mapping of stakeholders working with antimicrobial resistance. 2016.

16 Bramer WM, Giustini D, de Jonge GB, *et al.* De-duplication of database search results for systematic reviews in EndNote. *J Med Libr Assoc* 2016;**104**:240–3. doi:10.3163/1536-5050.104.3.014

17 Tricco AC, Lillie E, Zarin W, *et al.* PRISMA Extension for Scoping Reviews (PRISMA-ScR): Checklist and Explanation. *Ann Intern Med* 2018;**169**:467–73. doi:10.7326/M18-0850

For peer review only - http://bmjopen.bmj.com/site/about/guidelines.xhtml

Supplemental material

Supplementary Table 1. Full search terms for the bibliographic databases

Embase
http://www.embase.com

#		Results 10/12/2021
**1** – AMR	('drug resistance'/de OR 'antibiotic resistance'/exp OR 'antifungal resistance'/exp OR 'cross resistance'/exp OR 'extensive drug resistance'/exp OR 'multidrug resistance'/exp OR (drug-resistan* OR multidrug-resistan* OR anti-biotic-resistan* OR antibiotic-resistan* OR antibacterial-resistan* OR anti-bacterial-resistan* OR bacterial-resistan* OR bacterium-resistan* OR antimicrobial-resistan* OR microbial-resistan* OR anti-fungal-resistan* OR antifungal-resistan* OR cross-resistan* OR antibiotic-non-susceptib* OR antibiotic-nonsusceptib* OR drug-non-susceptib* OR drug-nonsusceptib*):ab,ti)<br>OR<br>('carbapenem resistant Acinetobacter baumannii'/exp OR ((Acinetobacter-baumannii* OR Achromobacter-mucosus OR Acinetobacter-baumanii OR Acinetobacter-baumanni OR Acinetobacter-genomosp.-2 OR Acinetobacter-genomospecies-2 OR Bacterium-anitratum OR Bact.-anitratum) AND (carbapenem-resistan* OR carbapenem-non-susceptib* OR carbapenem-nonsuscept*)):ti,ab)<br>OR<br>('carbapenem resistant Pseudomonas aeruginosa'/exp OR ((Pseudomonas-aeruginosa OR Bacillus-aeruginosus OR Bacillus-pyocyaneus OR Bacterium-aeruginosum OR Bacterium-pyocyaneum OR blue-pus-organism OR Micrococcus-pyocyaneus OR P.-aeruginosa OR Pseudomonas-polycolor OR Pseudomonas-pyoceaneus OR Pseudomonas-pyocyanea OR Pseudomonas-pyocyaneus) AND (carbapenem-resistan* OR carbapenem-non-susceptib* OR carbapenem-nonsuscept*)):ti,ab)<br>OR<br>('carbapenem-resistant Enterobacteriaceae'/exp OR ((Enterobacteriaceae OR enteric-bacteria OR enterobacteria OR enterobacteriacea OR enterobacterium OR Klebsiella-pneumonia OR Escherichia-coli OR Bacillus-coli OR Bacillus-escherichii OR Bacterium-coli OR Bacterium-coli OR bacterium-E3 OR coli-bacillus OR coli-bacterium OR colibacillus OR colon-bacillus OR E-coli OR E.-coli OR E.coli OR Enterococcus-coli OR Escherichia-alkalescens OR Enterobacter OR Serratia OR Proteus OR Providencia OR Morganella) AND (carbapenem-resistan* OR carbapenem-non-susceptib* OR carbapenem-nonsuscept* OR 3rd-generation-cephalosporin-resistan* OR third-generation-cephalosporin-resistan* OR third-generation-cephalosporin-non-susceptib*)):ti,ab)<br>OR	513,168

For peer review only

	('vancomycin resistant Enterococcus'/exp OR (VAN-R-enterococci OR VAN-R-Enterococcus OR (Streptococcus-faecium OR Enterococcus-faecium) AND (vancomycin-resistan* OR VAN-resistan* OR vancomycin-non-susceptib* OR vancomycin-nonsusceptib*)):ti,ab)<br>OR<br>('methicillin resistant Staphylococcus aureus'/exp OR 'methicillin resistant Staphylococcus aureus infection'/exp OR 'vancomycin resistant Staphylococcus aureus'/exp OR 'vancomycin intermediate Staphylococcus aureus'/exp OR (MRSA OR VRSA OR (Staphylococcus-aureus OR S.-aureus) AND (vancomycin-resistan* OR VAN-resistan* OR vancomycin-nonsusceptib* OR vancomycin-non-susceptib* OR Van-intermediate-resistan* OR vancomycin-intermediate-resistan* OR methicillin-resistan* OR methicillin-nonsusceptib* OR methicillin-non-susceptib*)):ti,ab)<br>OR<br>(((Helicobacter-pylori OR Campylobacter-pylori* OR Helicobacter-nemestrinae) AND (clarithromycin-resistan* OR clarithromycin-nonsusceptible OR clarithromycin-non-susceptib*)):ti,ab)<br>OR<br>(((Campylobacter) AND (fluoroquinolone-resistan* OR fluorinated-quinolone-resistan* OR fluoro-quinolone-resistan* OR fluoroquinolones-resistan* OR fluoroquinolone-nonsusceptible OR fluoroquinolone-non-susceptible)):ti,ab)<br>OR<br>(((Salmonella) AND (fluoroquinolone-resistan* OR fluorinated-quinolone-resistan* OR fluoro-quinolone-resistan* OR fluoroquinolones-resistan* OR fluoroquinolone-nonsusceptible OR fluoroquinolone-non-susceptible)):ti,ab)<br>OR<br>(((Neisseria-gonorrhoeae OR Gonococcus-neisseri OR Gonococcus-neisseria OR Micrococcus-gonorrhoeae OR N.-gonorrhoeae OR Neisseria-gonococcus OR Neisseria-gonorrhaeae OR Neisseria-gonorrhea OR Neisseria-gonorrheae OR Neisseria-gonorrhoea) AND (3rd-generation-cephalosporin-resistan* OR third-generation-cephalosporin-resistan* OR fluoroquinolone-resistan* OR fluorinated-quinolone-resistan* OR fluoro-quinolone-resistan* OR fluoroquinolones-resistan* OR fluoroquinolone-nonsusceptible OR fluoroquinolone-non-susceptible)):ti,ab)<br>OR<br>('penicillin resistant Streptococcus pneumoniae'/exp OR ((Streptococcus-pneumoniae OR Diplococcus-pneumoniae OR Micrococcus-pneumoniae OR Pneumococcus OR pneumococcus OR Pneumococcus-pneumoniae) AND (penicillin-resistan* OR penicillin-non-susceptib* OR penicillin-nonsusceptib* OR fluoroquinolone-nonsusceptible OR fluoroquinolone-non-susceptible)):ti,ab)<br>OR	

For peer review only

	(((Haemophilus-influenza* OR Hemophilus-influenza* OR H.-influenza* OR Bacterium-influenza* OR Coccobacillus-pfeifferi OR Hemophilus-influenzae OR influenza-bacillus OR Mycobacterium-influenzae OR pfeiffer-bacillus) AND (ampicillin-resistan* OR Amp-resistan* OR ampicillin-nonsusceptib* OR ampicillin-non-susceptib*)):ti,ab)<br>OR<br>(((Shigella) AND (fluoroquinolone-resistan* OR fluorinated-quinolone-resistan* OR fluoro-quinolone-resistan* OR fluoroquinolones-resistan* OR fluoroquinolone-nonsusceptible OR fluoroquinolone-non-susceptible)):ti,ab)<br>OR<br>('multidrug resistant tuberculosis'/exp OR (RR-TB OR MDR-TB OR Pre-XDR-TB OR XDR-TB OR drug-resistant-TB OR multidrug-resistant-TB OR multidrug-resistant-tuberculos* OR drug-resistant-tuberculos* OR ((Mycobacterium-tuberculos* OR pulmonary-tuberculos* OR pulmonary-TB) AND (isoniazid-resistan* OR rifampicin-resistan* OR floroquinolone-resistan* OR levofloxacin-resistan* OR moxifloxacin-resistan* OR bedaquiline-resistan* OR linezolid-resistan* OR multidrug-resistan* OR drug-resistan*))):ab,ti)<br>OR<br>(((Aspergillus OR a.-fumigatus OR a.-flavus OR Neosartorya-fumigate OR Candida OR c.-auris OR c.-albicans) AND (resistan* OR non-susceptib* OR nonsusceptib*)):ti,ab)	
**#2 -** SR	('systematic review'/de OR 'meta-analysis'/de OR ((systematic* NEAR/3 review*) OR metaanaly* OR meta-analy* OR scoping):ab,ti)	564,680
**#3**	#1 AND #2	7,288
**#4 –** Animal Studies filter	NOT ('animal'/exp OR 'nonhuman'/exp NOT ('animal'/exp OR 'nonhuman'/exp AND 'human'/exp))	6,949
**#5** Specific animals	NOT (rat OR rats OR mouse OR mice OR hamster OR hamsters OR dog OR dogs OR cat OR cats OR bovine OR sheep):ti	6946
**#6-** Date filter	#5 AND [1-1-2012]/sd NOT [2-1-2022]/sd AND [2012-2022]/py	5,288

Pubmed
https://pubmed.ncbi.nlm.nih.gov/

#		**Results 10/12/2021**
**1** – AMR	("Drug Resistance, Bacterial"[Mesh] OR "Drug Resistance, Fungal"[Mesh] OR "Drug Resistance, Multiple, Bacterial"[Mesh] OR "Drug Resistance, Multiple, Fungal"[Mesh] OR "Antimicrobial Stewardship"[Mesh] OR drug-resistan*[tiab] OR multidrug-	318,242

	resistan*[tiab] OR anti-biotic-resistan*[tiab] OR antibiotic-resistan*[tiab] OR antibacterial-resistan*[tiab] OR anti-bacterial-resistan*[tiab] OR bacterial-resistan*[tiab] OR bacterium-resistan*[tiab] OR antimicrobial-resistan*[tiab] OR microbial-resistan*[tiab] OR anti-fungal-resistan*[tiab] OR antifungal-resistan*[tiab] OR cross-resistan*[tiab] OR antibiotic-non-susceptib*[tiab] OR antibiotic-nonsusceptib*[tiab] OR drug-non-susceptib*[tiab] OR drug-nonsusceptib*[tiab])<br>OR<br>((("Acinetobacter baumannii"[Mesh] OR Acinetobacter-baumannii*[tiab] OR Achromobacter-mucosus[tiab] OR Acinetobacter-baumanii[tiab] OR Acinetobacter-baumanni[tiab] OR Acinetobacter-genomosp.-2[tiab] OR Acinetobacter-genomospecies-2[tiab] OR Bacterium-anitratum[tiab] OR Bact.-anitratum[tiab]) AND (carbapenem-resistan*[tiab] OR carbapenem-non-susceptib*[tiab] OR carbapenem-nonsuscept*[tiab])))<br>OR<br>((("Pseudomonas aeruginosa"[Mesh] OR Pseudomonas-aeruginosa[tiab] OR Bacillus-aeruginosus[tiab] OR Bacillus-pyocyaneus[tiab] OR Bacterium-aeruginosum[tiab] OR Bacterium-pyocyaneum[tiab] OR blue-pus-organism[tiab] OR Micrococcus-pyocyaneus[tiab] OR P.-aeruginosa[tiab] OR Pseudomonas-polycolor[tiab] OR Pseudomonas-pyoceaneus[tiab] OR Pseudomonas-pyocyanea[tiab] OR Pseudomonas-pyocyaneus[tiab]) AND (carbapenem-resistan*[tiab] OR carbapenem-non-susceptib*[tiab] OR carbapenem-nonsuscept*[tiab])))<br>OR<br>(("Carbapenem-Resistant Enterobacteriaceae"[Mesh] OR ("Enterobacteriaceae"[Mesh] OR Enterobacteriaceae[tiab] OR enteric-bacteria[tiab] OR enterobacteria[tiab] OR enterobacteriacea[tiab] OR enterobacterium[tiab] OR Klebsiella-pneumonia[tiab] OR Escherichia-coli[tiab] OR Bacillus-coli[tiab] OR Bacillus-escherichii[tiab] OR Bacterium-coli[tiab] OR Bacterium-coli[tiab] OR bacterium-E3[tiab] OR coli-bacillus[tiab] OR coli-bacterium[tiab] OR colibacillus[tiab] OR colon-bacillus[tiab] OR E-coli[tiab] OR E.-coli[tiab] OR E.coli[tiab] OR Enterococcus-coli[tiab] OR Escherichia-alkalescens[tiab] OR Enterobacter[tiab] OR Serratia[tiab] OR Proteus[tiab] OR Providencia[tiab] OR Morganella[tiab]) AND (carbapenem-resistan*[tiab] OR carbapenem-non-susceptib*[tiab] OR carbapenem-nonsuscept*[tiab] OR 3rd-generation-cephalosporin-resistan*[tiab] OR third-generation-cephalosporin-resistan*[tiab] OR third-generation-cephalosporin-non-susceptib*[tiab])))<br>OR<br>("Vancomycin-Resistant Enterococci"[Mesh] OR VAN-R-enterococci OR VAN-R-Enterococcus OR<br>(("Enterococcus faecium"[Mesh] OR Streptococcus-faecium OR Enterococcus-faecium) AND ("Vancomycin Resistance"[Mesh] OR	

For peer review only

	vancomycin-resistan*[tiab] OR VAN-resistan*[tiab] OR vancomycin-non-susceptib*[tiab] OR vancomycin-nonsusceptib*[tiab])))<br>OR<br>("Methicillin-Resistant Staphylococcus aureus"[Mesh] OR "vancomycin resistant Staphylococcus aureus"[mesh] OR "Vancomycin-Resistant Staphylococcus aureus"[Mesh] OR MRSA[tiab] OR VRSA[tiab] OR (("Staphylococcus aureus"[Mesh] OR Staphylococcus-aureus[tiab] OR S.-aureus[tiab]) AND ("Methicillin Resistance"[Mesh] OR "Vancomycin Resistance"[Mesh] OR vancomycin-resistan*[tiab] OR VAN-resistan*[tiab] OR vancomycin-nonsusceptib*[tiab] OR vancomycin-non-susceptib*[tiab] OR Van-intermediate-resistan*[tiab] OR vancomycin-intermediate-resistan*[tiab] OR methicillin-resistan*[tiab] OR methicillin-nonsusceptib*[tiab])))<br>OR<br>((("Helicobacter pylori"[Mesh] OR Helicobacter-pylori[tiab] OR Campylobacter-pylori*[tiab] OR Helicobacter-nemestrinae) AND (clarithromycin-resistan* OR clarithromycin-non-susceptib*[tiab])))<br>OR<br>((("Campylobacter"[Mesh] OR Campylobacter[tiab]) AND (fluoroquinolone-resistan*[tiab] OR fluorinated-quinolone-resistan*[tiab] OR fluoro-quinolone-resistan*[tiab] OR fluoroquinolones-resistan*[tiab] OR fluoroquinolone-nonsusceptible[tiab] OR fluoroquinolone-non-susceptible[tiab])))<br>OR<br>((("Salmonella"[Mesh] OR Salmonella[tiab]) AND (fluoroquinolone-resistan*[tiab] OR fluorinated-quinolone-resistan*[tiab] OR fluoro-quinolone-resistan*[tiab] OR fluoroquinolones-resistan*[tiab] OR fluoroquinolone-nonsusceptible[tiab] OR fluoroquinolone-non-susceptible[tiab])))<br>OR<br>((("Neisseria gonorrhoeae"[Mesh] OR Neisseria-gonorrhoeae[tiab] OR Gonococcus-neisseri[tiab] OR Gonococcus-neisseria[tiab] OR Micrococcus-gonorrhoeae[tiab] OR N.-gonorrhoeae[tiab] OR Neisseria-gonococcus[tiab] OR Neisseria-gonorrhaeae[tiab] OR Neisseria-gonorrhea[tiab] OR Neisseria-gonorrheae[tiab] OR Neisseria-gonorrhoea[tiab]) AND (3rd-generation-cephalosporin-resistan*[tiab] OR third-generation-cephalosporin-resistan*[tiab] OR fluoroquinolone-resistan*[tiab] OR fluorinated-quinolone-resistan*[tiab] OR fluoro-quinolone-resistan*[tiab] OR fluoroquinolones-resistan*[tiab] OR fluoroquinolone-nonsusceptible[tiab] OR fluoroquinolone-non-susceptible[tiab])))<br>OR<br>((("Streptococcus pneumoniae"[Mesh] OR Streptococcus-pneumoniae[tiab] OR Diplococcus-pneumoniae[tiab] OR Micrococcus-pneumoniae[tiab] OR Pneumococcus[tiab] OR pneumococcus[tiab] OR Pneumococcus-pneumoniae[tiab]) AND	

For peer review only

	("Penicillin Resistance"[Mesh] OR penicillin-resistan*[tiab] OR penicillin-non-susceptib*[tiab] OR penicillin-nonsusceptib*[tiab]))) OR ((("Haemophilus influenzae"[Mesh] OR Hemophilus-influenza*[tiab] OR Bacterium-influenzae OR Coccobacillus-pfeifferi OR H.-influenza*[tiab] OR Haemophilus-influenza*[tiab] OR influenza-bacillus[tiab] OR Mycobacterium-influenzae[tiab] OR pfeiffer-bacillus[tiab]) AND (ampicillin-resistan*[tiab] OR Amp-resistan*[tiab] OR ampicillin-nonsusceptib*[tiab] OR ampicillin-non-susceptib*[tiab]))) OR ((("Shigella"[Mesh] OR Shigella[tiab]) AND (fluoroquinolone-resistan*[tiab] OR fluorinated-quinolone-resistan*[tiab] OR fluoro-quinolone-resistan*[tiab] OR fluoroquinolones-resistan*[tiab] OR fluoroquinolone-nonsusceptible[tiab] OR fluoroquinolone-non-susceptible[tiab]))) OR ("Tuberculosis, Multidrug-Resistant"[Mesh] OR "Extensively Drug-Resistant Tuberculosis"[Mesh] OR RR-TB OR MDR-TB OR Pre-XDR-TB OR XDR-TB OR drug-resistant-TB OR multidrug-resistant-TB OR multidrug-resistant-tuberculos*[tiab] OR drug-resistant-tuberculos*[tiab] OR ((Mycobacterium-tuberculos*[tiab] OR pulmonary-tuberculos*[tiab] OR pulmonary-TB[tiab]) AND (isoniazid-resistan*[tiab] OR rifampicin-resistan*[tiab] OR floroquinolone-resistan*[tiab] OR levofloxacin-resistan*[tiab] OR moxifloxacin-resistan*[tiab] OR bedaquiline-resistan*[tiab] OR linezolid-resistan*[tiab] OR multidrug-resistan*[tiab] OR drug-resistan*[tiab]))) OR ((("Aspergillus"[Mesh] OR Aspergillus[tiab] OR a.-fumigatus[tiab] OR a.-flavus[tiab] OR Neosartorya-fumigate[tiab] OR Candida[tiab] OR c.-auris[tiab] OR c.-albicans[tiab]) AND (resistan*[tiab] OR non-susceptib*[tiab] OR nonsusceptib*[tiab])))	
**#2 -** SR	"Systematic Reviews as Topic"[Mesh] OR "Systematic Review" [Pt] OR systematic[sb] OR systematic-review*[tiab] OR meta-analysis[pt] OR meta-analysis as topic[mh] OR metaanalys*[tiab] OR meta-analys*[tiab] OR scoping[tiab]	366,960
**#3**	#1 AND #2	3,887
**#4 –** Animal Studies filter	NOT ("Animals"[Mesh] NOT ("Animals"[Mesh] AND "Humans"[Mesh]))	3,845
**#5** Specific animals	NOT (rat[ti] OR rats[ti] OR mouse[ti] OR mice[ti] OR hamster[ti] OR hamsters[ti] OR dog[ti] OR dogs[ti] OR cat[ti] OR cats[ti] OR bovine[ti] OR sheep[ti])	3,839
**#6-** Date filter	#5 AND [1-1-2012]/sd NOT [2-1-2022]/sd AND [2012-2022]/py	3,181

For peer review only

Web of Science
https://www.webofscience.com/

#		Results 06/12/2021
**1** – AMR	(drug-resistan* OR multidrug-resistan* OR anti-biotic-resistan* OR antibiotic-resistan* OR antibacterial-resistan* OR anti-bacterial-resistan* OR bacterial-resistan* OR bacterium-resistan* OR antimicrobial-resistan* OR microbial-resistan* OR anti-fungal-resistan* OR antifungal-resistan* OR cross-resistan* OR antibiotic-non-susceptib* OR antibiotic-nonsusceptib* OR drug-non-susceptib* OR drug-nonsusceptib*)<br>OR<br>(((Acinetobacter-baumannii* OR Achromobacter-mucosus OR Acinetobacter-baumanii OR Acinetobacter-baumanni OR Acinetobacter-genomosp.-2 OR Acinetobacter-genomospecies-2 OR Bacterium-anitratum OR Bact.-anitratum) AND (carbapenem-resistan* OR carbapenem-non-susceptib* OR carbapenem-nonsuscept*)))<br>OR<br>(((Pseudomonas-aeruginosa OR Bacillus-aeruginosus OR Bacillus-pyocyaneus OR Bacterium-aeruginosum OR Bacterium-pyocyaneum OR blue-pus-organism OR Micrococcus-pyocyaneus OR P.-aeruginosa OR Pseudomonas-polycolor OR Pseudomonas-pyoceaneus OR Pseudomonas-pyocyanea OR Pseudomonas-pyocyaneus) AND (carbapenem-resistan* OR carbapenem-non-susceptib* OR carbapenem-nonsuscept*)))<br>OR<br>(((Enterobacteriaceae OR enteric-bacteria OR enterobacteria OR enterobacteriacea OR enterobacterium OR Klebsiella-pneumonia OR Escherichia-coli OR Bacillus-coli OR Bacillus-escherichii OR Bacterium-coli OR Bacterium-coli OR bacterium-E3 OR coli-bacillus OR coli-bacterium OR colibacillus OR colon-bacillus OR E-coli OR E.-coli OR E.coli OR Enterococcus-coli OR Escherichia-alkalescens OR Enterobacter OR Serratia OR Proteus OR Providencia OR Morganella) AND (carbapenem-resistan* OR carbapenem-non-susceptib* OR carbapenem-nonsuscept* OR 3rd-generation-cephalosporin-resistan* OR third-generation-cephalosporin-resistan* OR third-generation-cephalosporin-non-susceptib*)))<br>OR<br>(VAN-R-enterococci OR VAN-R-Enterococcus OR ((Streptococcus-faecium OR Enterococcus-faecium) AND (vancomycin-resistan* OR VAN-resistan* OR vancomycin-non-susceptib* OR vancomycin-nonsusceptib*)))<br>OR<br>(MRSA OR VRSA OR ((Staphylococcus-aureus OR S.-aureus) AND (vancomycin-resistan* OR VAN-resistan* OR vancomycin-	339,733

For peer review only

	nonsusceptib* OR vancomycin-non-susceptib* OR Van-intermediate OR vancomycin-intermediate OR methicillin-resistan*)))<br>OR<br>(((Helicobacter-pylori OR Campylobacter-pylori* OR Helicobacter-nemestrinae) AND (clarithromycin-resistan* OR clarithromycin-non-susceptib*)))<br>OR<br>(((Campylobacter) AND (fluoroquinolone-resistan* OR fluorinated-quinolone-resistan* OR fluoro-quinolone-resistan* OR fluoroquinolones-resistan* OR fluoroquinolone-nonsusceptible OR fluoroquinolone-non-susceptible)))<br>OR<br>(((Salmonella) AND (fluoroquinolone-resistan* OR fluorinated-quinolone-resistan* OR fluoro-quinolone-resistan* OR fluoroquinolones-resistan* OR fluoroquinolone-nonsusceptible OR fluoroquinolone-non-susceptible)))<br>OR<br>(((Neisseria-gonorrhoeae OR Gonococcus-neisseri OR Gonococcus-neisseria OR Micrococcus-gonorrhoeae OR N.-gonorrhoeae OR Neisseria-gonococcus OR Neisseria-gonorrhaeae OR Neisseria-gonorrhea OR Neisseria-gonorrheae OR Neisseria-gonorrhoea) AND (3rd-generation-cephalosporin-resistan* OR third-generation-cephalosporin-resistan* OR fluoroquinolone-resistan* OR fluorinated-quinolone-resistan* OR fluoro-quinolone-resistan* OR fluoroquinolones-resistan* OR fluoroquinolone-nonsusceptible OR fluoroquinolone-non-susceptible)))<br>OR<br>(((Streptococcus-pneumoniae OR Diplococcus-pneumoniae OR Micrococcus-pneumoniae OR Pneumococcus OR pneumococcus OR Pneumococcus-pneumoniae) AND (penicillin-resistan* OR penicillin-non-susceptib* OR penicillin-nonsusceptib*)))<br>OR<br>(((Hemophilus-influenza* OR Bacterium-influenzae OR Coccobacillus-pfeifferi OR H.-influenza* OR Haemophilus-influenza* OR influenza-bacillus OR Mycobacterium-influenzae OR pfeiffer-bacillus) AND (ampicillin-resistan* OR Amp-resistan* OR ampicillin-nonsusceptib* OR ampicillin-non-susceptib*)))<br>OR<br>(((Shigella) AND (fluoroquinolone-resistan* OR fluorinated-quinolone-resistan* OR fluoro-quinolone-resistan* OR fluoroquinolones-resistan* OR fluoroquinolone-nonsusceptible OR fluoroquinolone-non-susceptible)))<br>OR<br>(RR-TB OR MDR-TB OR Pre-XDR-TB OR XDR-TB OR drug-resistant-TB OR multidrug-resistant-TB OR multidrug-resistant-tuberculos* OR drug-resistant-tuberculos* OR ((Mycobacterium-tuberculos* OR pulmonary-tuberculos* OR pulmonary-TB) AND	

For peer review only

	(isoniazid-resistan* OR rifampicin-resistan* OR floroquinolone-resistan* OR levofloxacin-resistan* OR moxifloxacin-resistan* OR bedaquiline-resistan* OR linezolid-resistan* OR multidrug-resistan* OR drug-resistan*)))<br>OR<br>(((Aspergillus OR a.-fumigatus OR a.-flavus OR Neosartorya-fumigate OR Candida OR c.-auris OR c.-albicans) AND (resistan* OR non-susceptib*)))	
**#2 -** SR	TS=("Systematic Review" OR (systematic NEAR/3 *review*) OR metaanalys* OR meta-analys* OR met*analys* OR (scoping NEAR/3 (literature OR *review*)))	602,250
**#3**	#1 AND #2	5,279
**#4** Specific animals	NOT TI=(rat OR rats OR mouse OR mice OR hamster OR hamsters OR dog OR dogs OR cat OR cats OR bovine OR sheep)	5,254
**#5-** Date filter	#5 AND Timespan: 2012-01-01 to 2022-01-01 (Index Date)	4,428

For peer review only

Supplementary Table 2. Data attributes to be extracted

**Document-level attributes**	
Author(s);	
Author type	- Government agency;<br>- International (technical) agency/organization;<br>- Funding agency/donor;<br>- Research organization/university;<br>- Expert group/professional association;<br>- Individual authors;<br>- Audit/accreditation body;<br>- Private for-profit organization;<br>- Non-government organization;<br>- Other<br>- Unassigned
Journal/Book	Open text
Publisher	Open text
Publication year	
Country/countries of origin	Open text
Funding source	Open text
Information source	- WHO document;<br>- WHO guideline;<br>- Grey literature;<br>- Systematic reviews in the bibliographic database;<br>- Scientific papers suggested by the WHO AMR Steering Group
Purpose/Type of document	- Guidance/guideline;<br>- Report;<br>- Systematic review/meta-analysis;<br>- Narrative review/commentary/editorial;<br>- Other
Data extractor 1 and 2	
Date of data extraction 1 and 2	
Confirmation on document's inclusion/exclusion criteria	Document inclusion criteria:<br>1. Document describes one or more knowledge gaps or research questions on AMR, including priorities, framework, components, elements, or steps for the description (i.e., epidemiology, burden and drivers), delivery, development and/or discovery of tools, products, or interventions for AMR prevention, diagnosis and/or care & treatment.<br>2. Document has a global or regional application and/or relevance for LMIC.<br>Document exclusion criteria:

For peer review only

	1. Document only describes individual research studies or case reports;<br>2. Document is not related to bacteria included in the WHO priority pathogen list, M. tuberculosis or critical fungi (e.g., parasites, viruses);<br>3. Document is related to AMR in the non-human sectors;<br>4. Document has no identifiable authors, publisher and year of publication.
**Document-level and research-question level attributes**	
Geographical scope	- Unassigned<br>- Global<br>- Regional;<br>- National;<br>- Subnational/local
Region (if applicable)	Open text;
Country/-ies (if applicable)	Open text;
Resource/socioeconomic context (World Bank definition)	- Unassigned<br>- General/overall<br>- LIC<br>- MIC<br>- HIC<br>- Low- and Middle-Income Countries (LMIC)
Population	- Unassigned<br>- General/overall<br>- Adult<br>- Children<br>- Neonates<br>- Elderly<br>- Immunocompromised<br>- Comorbidities<br>- Pregnant women<br>- Other vulnerable groups
Setting	- Unassigned<br>- General/overall<br>- Community<br>- Health care system<br>○ Primary health care<br>○ Acute-care hospitals (including secondary and tertiary care)<br>○ Long-term care facilities (including nursing homes)<br>○ ICU
Micro-organism group	- Unassigned<br>- General/overall<br>- PPL<br>- TB

For peer review only

	- Critical fungi
Antibiotic-microorganism combination (refer to Table 1)<br>-	- Unassigned<br>- General/overall<br>- Acinetobacter baumannii: carbapenem-resistant<br>- Pseudomonas aeruginosa: carbapenem-resistant<br>- Enterobacteriaceae (including Klebsiella pneumonia, Escherichia coli, Enterobacter spp., Serratia spp., Proteus spp., and Providencia spp, Morganella spp.): carbapenem-resistant, 3rd generation cephalosporin-resistant<br>- Enterococcus faecium: vancomycin-resistant<br>- Staphylococcus aureus: methicillin-resistant, vancomycin-intermediate and -resistant<br>- Helicobacter pylori: clarithromycin-resistant<br>- Campylobacter: fluoroquinolone-resistant<br>- Salmonella spp.: fluoroquinolone-resistant<br>- Neisseria gonorrhoeae: 3rd generation cephalosporin-resistant, fluoroquinolone-resistant<br>- Streptococcus pneumoniae: penicillin-non-susceptible<br>- Haemophilus influenzae: ampicillin-resistant<br>- Shigella spp.: fluoroquinolone-resistant<br>- RR-TB<br>- MDR-TB<br>- Pre-XDR-TB<br>- XDR-TB<br>- Aspergillus spp (including fumigatus and flavus)<br>- Candida spp. (including albicans and auris).
Syndrome	- Unassigned<br>- General/overall<br>- Bloodstream infection/sepsis<br>- Pneumonia/respiratory tract<br>- Gastrointestinal infection<br>- Urinary tract infection<br>- Skin and soft tissue<br>- Central nervous system infection<br>- Sexually Transmitted Infection<br>- TB-general/unspecified<br>- TB-pulmonary<br>- TB-extrapulmonary<br>- Other
Sector	- Human health sector<br>- One health (intersection/overlapping between animal, environment and human health sector)
Topic	- Unassigned<br>- General/overall<br>- AMR National Action Plans and Monitoring & Evaluation<br>- AMR surveillance<br>- Antimicrobial consumption surveillance

For peer review only

	- AMR One Health<br>- Antimicrobial Use/AMR Stewardship/Essential Medicines<br>- Antimicrobial R&D<br>- AMR awareness<br>- Diagnostics (including R&D)<br>- Infection Prevention and Control<br>- Water, Sanitation, Hygiene and Health<br>- Immunization/Vaccine (including Product & Delivery Research)<br>- Food safety/food borne diseases<br>- Sexually Transmitted Infections<br>- TB Prevention, Diagnosis, Treatment, Care & Innovation<br>- Newborn health<br>- Health systems strengthening/Universal Health Coverage/Policy and regulatory
**Research-question level attributes**	
Theme	- Prevention (subcategories for CAI and HAI, immunization, IPC, other)<br>- Diagnosis<br>- Care & treatment (subcategories: CAI and HAI)<br>- Unassigned
Domain	- Descriptive (subcategories: measuring the burden; understanding risk factors; measuring prevalence of exposure to risk factors; Evaluating the efficacy and effectiveness of interventions in place; measuring prevalence of coverage of interventions in place)<br>- Delivery (subcategories: Health policy analysis; Health system structure analysis; Financing/costs analysis; Human resources; Provision/infrastructure; Operations research; Responsiveness/recipients)<br>- Development (subcategories: acceptability, affordability, deliverability/feasibility, sustainability)<br>- Discovery (subcategories: medicines, diagnostics, vaccines, digital health technology, other preventive tools, other)<br>- Unassigned
Area	- Epidemiology (including drivers, prevalence and burden)<br>- Pharmacological and clinical<br>- Behavioural and social science<br>- Health economics/cost-effectiveness<br>- Legislation and regulation

Abbreviations: AMR: antimicrobial resistance; CAI: community-acquired infection; HAI: healthcare-associated infection; HIC: high-income country; ICU: intensive care unit; IPC: infection prevention and control; LIC: low-income country; LMIC: low- and middle-income country; MIC: middle-income country MDR-TB: multidrug-resistant tuberculosis; PPL: WHO priority pathogen list; Pre-XDR-TB: pre-extensively drug-resistant tuberculosis; RR-TB:

For peer review only

rifampicin-resistant tuberculosis; R&D: research and development; TB: tuberculosis; XDR-TB: extensively drug-resistant tuberculosis

For peer review only

Preferred Reporting Items for Systematic reviews and Meta-Analyses extension for Scoping Reviews (PRISMA-ScR) Checklist

SECTION	ITEM	PRISMA-ScR CHECKLIST ITEM	REPORTED ON PAGE #
**TITLE**			
Title	1	Identify the report as a scoping review.	1
**ABSTRACT**			
Structured summary	2	Provide a structured summary that includes (as applicable): background, objectives, eligibility criteria, sources of evidence, charting methods, results, and conclusions that relate to the review questions and objectives.	2
**INTRODUCTION**			
Rationale	3	Describe the rationale for the review in the context of what is already known. Explain why the review questions/objectives lend themselves to a scoping review approach.	5
Objectives	4	Provide an explicit statement of the questions and objectives being addressed with reference to their key elements (e.g., population or participants, concepts, and context) or other relevant key elements used to conceptualize the review questions and/or objectives.	5
**METHODS**			
Protocol and registration	5	Indicate whether a review protocol exists; state if and where it can be accessed (e.g., a Web address); and if available, provide registration information, including the registration number.	This paper is the review protocol
Eligibility criteria	6	Specify characteristics of the sources of evidence used as eligibility criteria (e.g., years considered, language, and publication status), and provide a rationale.	8-10, 13
Information sources*	7	Describe all information sources in the search (e.g., databases with dates of coverage and contact with authors to identify additional sources), as well as the date the most recent search was executed.	11
Search	8	Present the full electronic search strategy for at least 1 database, including any limits used, such that it could be repeated.	Supplemental material, Table S1
Selection of sources of evidence†	9	State the process for selecting sources of evidence (i.e., screening and eligibility) included in the scoping review.	12-14
Data charting process‡	10	Describe the methods of charting data from the included sources of evidence (e.g., calibrated forms or forms that have been tested by the team before their use, and whether data charting was done independently or in duplicate) and any processes for obtaining and confirming data from investigators.	13-14
Data items	11	List and define all variables for which data were sought and any assumptions and simplifications made.	Supplemental material, Table S2
Critical appraisal of individual sources of evidence§	12	If done, provide a rationale for conducting a critical appraisal of included sources of evidence; describe	14-15

For peer review only

SECTION	ITEM	PRISMA-ScR CHECKLIST ITEM	REPORTED ON PAGE #
		the methods used and how this information was used in any data synthesis (if appropriate).	
Synthesis of results	13	Describe the methods of handling and summarizing the data that were charted.	14
**RESULTS**			
Selection of sources of evidence	14	Give numbers of sources of evidence screened, assessed for eligibility, and included in the review, with reasons for exclusions at each stage, ideally using a flow diagram.	NA
Characteristics of sources of evidence	15	For each source of evidence, present characteristics for which data were charted and provide the citations.	NA
Critical appraisal within sources of evidence	16	If done, present data on critical appraisal of included sources of evidence (see item 12).	NA
Results of individual sources of evidence	17	For each included source of evidence, present the relevant data that were charted that relate to the review questions and objectives.	NA
Synthesis of results	18	Summarize and/or present the charting results as they relate to the review questions and objectives.	NA
**DISCUSSION**			
Summary of evidence	19	Summarize the main results (including an overview of concepts, themes, and types of evidence available), link to the review questions and objectives, and consider the relevance to key groups.	NA
Limitations	20	Discuss the limitations of the scoping review process.	3
Conclusions	21	Provide a general interpretation of the results with respect to the review questions and objectives, as well as potential implications and/or next steps.	NA
**FUNDING**			
Funding	22	Describe sources of funding for the included sources of evidence, as well as sources of funding for the scoping review. Describe the role of the funders of the scoping review.	16

JBI = Joanna Briggs Institute; PRISMA-ScR = Preferred Reporting Items for Systematic reviews and Meta-Analyses extension for Scoping Reviews.

* Where *sources of evidence* (see second footnote) are compiled from, such as bibliographic databases, social media platforms, and Web sites.

† A more inclusive/heterogeneous term used to account for the different types of evidence or data sources (e.g., quantitative and/or qualitative research, expert opinion, and policy documents) that may be eligible in a scoping review as opposed to only studies. This is not to be confused with *information sources* (see first footnote).

‡ The frameworks by Arksey and O'Malley (6) and Levac and colleagues (7) and the JBI guidance (4, 5) refer to the process of data extraction in a scoping review as data charting.

§ The process of systematically examining research evidence to assess its validity, results, and relevance before using it to inform a decision. This term is used for items 12 and 19 instead of "risk of bias" (which is more applicable to systematic reviews of interventions) to include and acknowledge the various sources of evidence that may be used in a scoping review (e.g., quantitative and/or qualitative research, expert opinion, and policy document).

From: Tricco AC, Lillie E, Zarin W, O'Brien KK, Colquhoun H, Levac D, et al. PRISMA Extension for Scoping Reviews (PRISMAScR): Checklist and Explanation. Ann Intern Med. 2018;169:467–473. doi: 10.7326/M18-0850.

For peer review only - http://bmjopen.bmj.com/site/about/guidelines.xhtml
